# Heat transfer analysis of fractional model of couple stress Casson tri-hybrid nanofluid using dissimilar shape nanoparticles in blood with biomedical applications

**DOI:** 10.1038/s41598-022-25127-z

**Published:** 2023-03-21

**Authors:** Muhammad Arif, Luca Di Persio, Poom Kumam, Wiboonsak Watthayu, Ali Akgül

**Affiliations:** 1 Fixed Point Research Laboratory, Fixed Point Theory and Applications Research Group, Center of Excellence in Theoretical and Computational Science (TaCS-CoE), Faculty of Science, King Mongkut’s University of Technology Thonburi (KMUTT), 126 Pracha Uthit Rd., Bang Mod, Thung Khru, Bangkok, 10140 Thailand; 2 Center of Excellence in Theoretical and Computational Science (TaCS-CoE), Faculty of Science, King Mongkut’s University of Technology Thonburi (KMUTT), 126 Pracha Uthit Rd., Bang Mod, Thung Khru, Bangkok, 10140 Thailand; 3 Department of Computer Science, College of Mathematics, University of Verona, Verona, Italy; 4 Department of Medical Research, China Medical University Hospital, China Medical University, Taichung, 40402 Taiwan; 5 Department of Mathematics, Faculty of Arts and Sciences, Siirt University, 56100 Siirt, Turkey; 6 Department of Computer Science and Mathematics, Lebanese American University, Beirut, Lebanon; 7 Mathematics Research Center, Department of Mathematics, Near East University, Near East Boulevard, PC: 99138, Nicosia /Mersin 10, Nicosia /Mersin, Turkey

**Keywords:** Biotechnology, Materials science, Mathematics and computing, Nanoscience and technology

## Abstract

During last decades the research of nanofluid is of great interest all over the World, particularly because of its thermal applications in engineering, and biological sciences. Although nanofluid performance is well appreciate and showed good results in the heat transport phenomena, to further improve conventional base fluids thermal performance an increasing number of researchers have started considering structured nanoparticles suspension in one base fluid. As to make an example, when considering the suspension of three different nanoparticles in a single base fluid we have the so called “ternary hybrid nanofluid”. In the present study three different shaped nanoparticles are uniformly dispersed in blood. In particular, the three different shaped nanoparticles are spherical shaped ferric oxide $\text{Fe}_{3} \text{O}_{4}$, platelet shaped zinc $\left( \text{Zn} \right)$, and cylindrical shaped gold $\left( \text{Au} \right)$, which are considered in blood base fluid because of related advance pharmaceutical applications. Accordingly, we focused our attention on the sharp evaluation of heat transfer for the unsteady couple stress Casson tri-hybrid nanofluid flow in channel. In particular, we formulated the problem via momentum and energy equations in terms of partial differential equations equipped with realistic physical initial and boundary conditions. Moreover, we transformed classical model into their fractional counterparts by applying the Atangana–Baleanu time-fractional operator. Solutions to velocity and temperature equations have been obtained by using both the Laplace and the Fourier transforms, while the effect of physical parameters on velocity and temperature profiles, have been graphically analyzed exploiting MATHCAD. In particular, latter study clearly shows that for higher values of volume fraction $\phi_{hnf}$ of the nanoparticles the fluid velocity declines, while the temperature rises for the higher values of volume fraction $\phi_{hnf}$ of the nanoparticles. Using blood-based ternary hybrid nanofluid enhances the rate of heat transfer up-to 8.05%, spherical shaped $\text{Fe}_{3} \text{O}_{4}$ enhances up-to 4.63%, platelet shaped $\left( \text{Zn} \right)$ nanoparticles enhances up-to 8.984% and cylindrical shaped gold $\left( \text{Au} \right)$ nanoparticles enhances up-to 10.407%.

## Introduction

One of the main challenges within the current Science panorama is the one to obtain concrete enhancements concerning the rate of heat transfer of regular base fluids. Therefore an increasing number of researchers has been attracted to investigate thermal properties of solid particles within conventional base fluids analysis.

To improve the thermal properties of the conventional fluid Choi^1^ was the first who gave the concept of dispersion of nano-meter particles in the base fluid. The obtained fluid after dispersion of particles is known as nanofluid and it shows promising thermal performance as compared to regular fluids. This idea has then been used in many fields of sciences and technology as, e.g., cooling system, electronic circuits, different heat exchanger systems, automotive thermal system with good thermal characteristics, and temperature reduction systems, accurate working capability of base fluids, cancer therapy, drug delivery and long-life span. Such experiments also shown that nanoparticles suspension in the regular fluid make better thermal performance of the base fluid with good stability in comparison to the fluid having milli and micro-sized solid particles. In particular, it is worth mentioning the works by Ali et al.^2^ about thermal performance of molybdenum disulfide $\text{MoS}_{2}$ nanoparticles in polyethylene glycol with thermal radiation, chemical reaction and ramped wall temperature, Wang et al.^3^ concerning the efficiency of nanoparticles in blood for the treatment of brain tumor, Fullstone et al.^4^, where the authors studied nanoparticles transport modelling using the blood flow also discussing shape, size and surface of nanoparticles then explaining how to improve the performance in biological systems. Within the huge scenario of physical applications, researchers taken different nanoparticles for many reasons, trying to exploit the specific nanoparticles characteristics to optimize provided solutions accordingly. Indeed, we can mention the works by Cótica et al.^5^ concerning the exploitation of the peculiar properties of magnetite $\text{Fe}_{3} \text{O}_{4}$ nanoparticles in blood, Jamil et al.^6^, where the authors examined the unsteady flow of blood with nanoparticles in the presence of periodic body acceleration, Feng et al.^7^, concerning relevant biological applications of Zinc oxide $\left( \text{Zn} \right)$ nanoparticles in blood, Bejawada et al.^8^ with respect to the study of viscous dissipative flow of nanofluid by using the Galerkin FEM method, Khan et al.^9^, concerning the investigation of magnetohydrodynamics 3D flow (KLL) Correlation and discuss their studies in real life applications and Gowda et al.^10^, where the authors derived computational model of nanofluid flow using curved stretching sheet and for solutions considered KKL method and modified model for heat flux. In addition to this there are variety of research have been carried out due increasing demand of the nanotechnology. Therefore, motivated from these applications some of them are discussed here in refs.^11–16^.

It is relevant to underline that the dispersion of single nanoparticles is often not enough to obtain required heat transfer rate, which implies, although its theoretical relevance, a fundamental lack concerning concrete analysis and solutions of both engineering and biological sciences applications.

That is the main reason why “hybrid nanofluid” approaches have been developed. Let us remark that hybrid nanofluids are obtained by the suspension of two or more than two nanoparticles which are then mixed in one single conventional base fluid. This new class of nanofluid model produce efficient enhancement in the phenomena of heat transfer as witnessed by several applications in many cooling systems, heat turbines in industries, heat generators, drug delivery as well as concerning biological problems. Sarkar et al.^17^ explored the thermal applications of hybrid nanofluids and claimed that hybrid performance is better than nano liquids by comparing pressure drop characteristics to mono nanofluids. Such a results stimulates further investigations as, e.g., Jamshed et al.^18^ concerning the influence of thermal increment in the solar aircraft by taking the hybrid nanofluid model, Ouni et al.^19^, where the authors provided some advance applications of engine oil using the idea of hybrid nanofluid flow in parabolic trough type solar collector, Redouane et al.^20^, with respect to the analysis of the impact of entropy and Brinkman-Forchheimer model by considering the applications of hybrid nanofluid in a rotating cylinder, Jamshed et al.^21^, discussing the comparative analysis of hybrid nanofluid with mono nanofluids and considering the flow through the extending surface, John et al.^22^ and Khan et al.^23^, in relation to the applications of hybrid nanofluid flow, during the analysis the scholar investigated that the working capability of hybrid nanofluid is good as compared to mono nanofluid, Sidik et al.^24^, concerning some basic operations and methods for the preparation of hybrid nanofluid discuss some techniques with stability and explain some useful applications of hybrid nano-composite in modern science and technology, Varun et al.^25^, where a two phase flow over a stretched cylinder with the uniform dispersion of hybrid nanofluid has been derived and Li et al.^26^, concerning aluminum oxide and copper hybrid nanofluid flow dynamics also in connection with the effect of entropy generation. Hosseinzadeh et al.^27^, studied heat transfer applications using hybrid nanofluid in various thermal systems. Salehi et al.^28^ developed the research of hydrothermal investigation of MHD squeezing hybrid nanofluid between two parallel plates. Zangooee et al.^29^ discussed the investigations of three dimensional hybrid nanofluid where the fluid is affected by the non-uniform MHD suing stretching shrinking sheet. Similarly, Hosseinzadeh et al.^30^, studied the suspension of hybrid nanoparticles over a vertical cylinder by choosing different shape nanoparticles and its impact on the heat transfer rate. Recently, Acharya^31,32^ studied some advance and unique applications of hybrid nanofluid in various physical circumstances. During the analysis of hybrid nanofluid flow it is observed that suspending two nanoparticles in a single base fluid at the same time in single base fluid is more effective compared to simple nanofluid. Some other theoretical and physical description of the hybrid nanofluid can be found in^33–35^.

It is worth stressing that fluid’s thermal properties strictly depend on the size and shape of the suspending nanoparticles, as shown by ,e.g., Sahu and Sarkar^36^ where the authors describe the influence of shape factor of nanoparticles in the flow and found some interesting advance applications in modern technology, Timofeeva et al.^37^ discussed and highlight the influence of nanoparticles on the flow and the dynamic viscosity of alumina nanofluid at different values. Similarly, different shaped of nano-meter sized particles dynamics have been analyzed by Jiang et al.^38^, discovering the shape factor of nanoparticles in their study and developed some advance cooling applications by considering the different size and shape nanoparticles. Sadaf et al.^39^ where the authors highlighted the new unique applications of tri-hybrid nanofluid choosing blood as base fluid and incorporating different shaped of nanoparticles and get some useful results.

The case of tri-hybrid nanofluid is realized by considering the uniform dispersion of three nanoparticles with various shapes dissolve in a single base fluid which has many applications in modern science.

Exploiting latter approach Arif et al.^40^ investigated tri-hybrid nanofluids flow using different shaped nanometer sized solid particles for the purpose of heat transfer analysis of radiator, while Adun et al.^41^ developed advance class of nanofluid model for tri-hybrid nanofluid and discussed the modern approach of the tri-hybrid nanofluid. Furthermore, in this study they describe the dynamics of tri-hybrid nanofluids, synthesis stability, thermal properties of tri-hybrid nanofluids, environmental effects and their heat transfer applications, Sahoo and Kumar^42^ calculated a new correlation to highlight the viscosity of tri-hybrid nanofluids with modern applications, and Xuan et al.^43^ provided interesting results of tri-hybrid nanofluids and explain the sensitive performance of tri-hybrid nanofluid.

The theory of Newtonian and non-Newtonian fluids has been widely applied to analyses different fluids dynamics, highlighting how non-Newtonian ones can be successfully considered when dealing with real-world problems. In the latter class, we would like to underline the prominent role played by the Casson fluid. Indeed, such type of fluid is characterized by having stress and rate of strain non-linear correlated. Between studies based on such a fluid, let us recall the ones by Alotaibi et al.^44^, concerning Casson nanofluid flow via convective heated non-linear extended surface with viscous dissipation, Hussain et al.^45^, where the authors discussed electromagnetic Casson nanofluid flow with shape factor of nanoparticles over a stretched sheet, Jamshed et al.^46^, devoted to the Casson nanofluid model for the advance applications of solar collector, and Naveen et al.^47^, where the authors explain the Casson hybrid nanofluid flow over a moving rotating disc in a Dafrcy-Forchheimer porous medium.

Within previously depicted scenarios, an increasing relevance has been given to the possible applications of fractional calculus, together with the analysis of related fractional integro-differential operators, widely used for a heterogeneous set of fluid dynamics applications as well as concerning the analysis of many other dynamical systems. It is worth mentioning that fractional order derivatives are characterized by sensitive memory effect. Between possible fractional derivatives, let us recall the one of Riemann Liouville, Caputo, Caputo-Fabrizio and Atangana–Baleanu, each characterized by specific characteristics to be used accordingly to different tasks we have to solve. As to give some relevant examples, in Liu et al.^48^ the authors discussed applications of fractional calculus, investigating radial basis functions for fractional derivatives and their uses, Hilfer^49^ analyzed applications of fractional calculus in the field of physics and modern sciences, in Yang^50^ we can found the study fractional calculus applied to modern science and technology, while Saqib et al.^51^ analyzed the generalized Brinkman type fluid with carbon nanotubes and ramped heating effect, see also, e.g., the Atangana-Balenufractional derivatives in^52,53^.

The present work addressed the heat performance of blood-based tri-hybrid nanofluids with various shape of nanometer sized solid particles uniformly dispersed in blood. Various shaped e.g., platelet, spherical and cylindrical nanoparticles are mixed in the blood. More specifically, the present analysis shows that the proposed blood-based ternary hybrid nanofluid have useful applications, indeed: (i) ferric oxide Fe_3_O_4_ nanoparticles have biomedical applications in targeted anticancer drug delivery system, (ii) Zn nanoparticles use as preventive and therapeutic agent and increase the immune responses against COVID-19 infectious disease, and (iii) gold $\left( \text{Au} \right)$ nanoparticles have useful applications in the cancer therapy. Moreover, in this research we are concentrated to developed and formulate a new class of non-Newtonian fluid model namely, couple stress Casson fluid. The fluid are allowed to flow in channel with the addition of external pressure acting on the fluid. Moreover, to overcome one of the main drawbacks of classical models, namely their inability to explain the memory effect, we transformed them into their fractional counterparts by applying the Atangana–Baleanu time-fractional operator. First the problem is formulated and then by generalizing AB-fractional derivative is applied and addressed the impact of it on the fluid flow. The transform model is then solved by the applications of (Laplace & Fourier) transforms. The physical exploration of the new idea of tri-hybrid nanofluid is performed in view of biological applications. The physical impact of all parameters are illustrated through graphs. To complete our study, we have also provided an in-depth comparative analysis related to tri-hybrid nanofluid and unitary nanofluid, showing that the first approach overcomes the second.

## Mathematical modeling and solution of the problem

**Figure 1:**
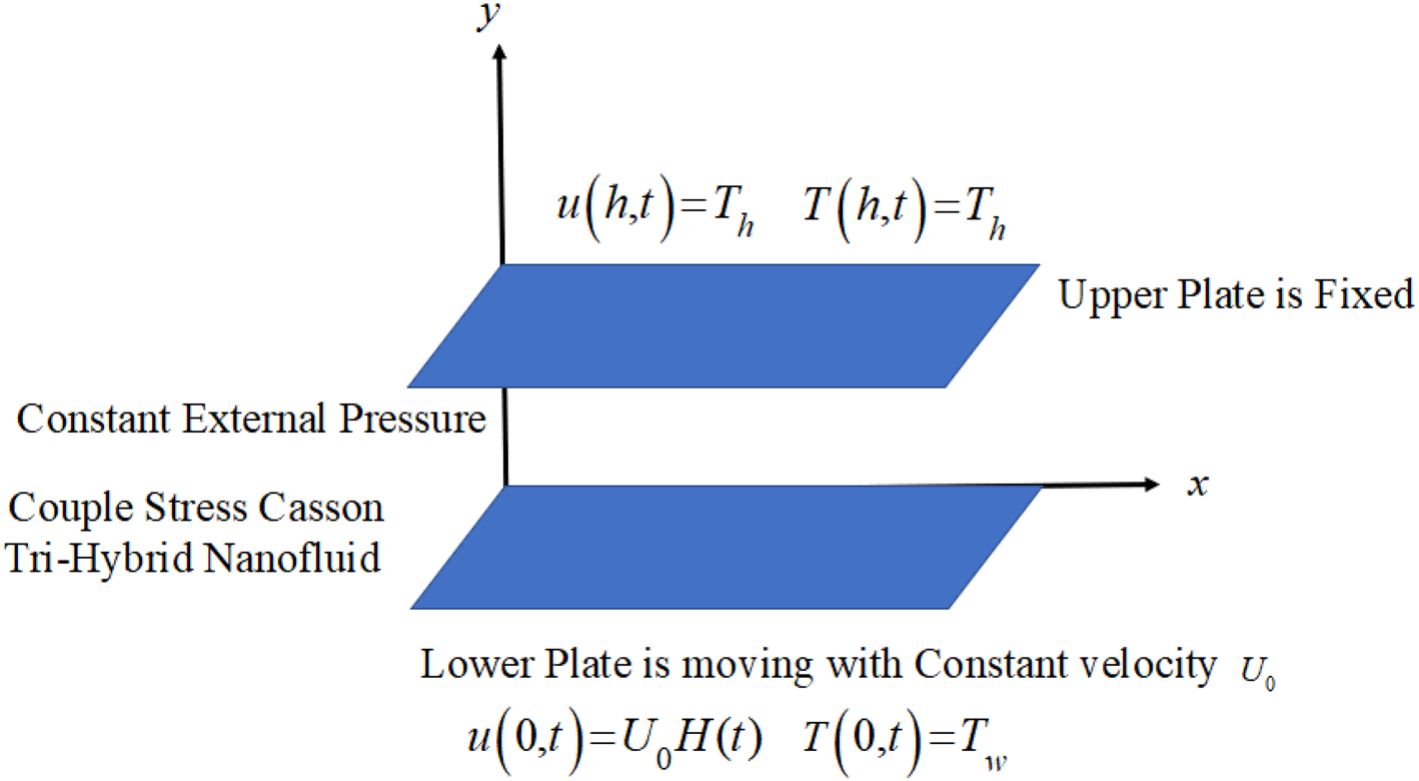
Geometry of the problem.

**Figure2:**
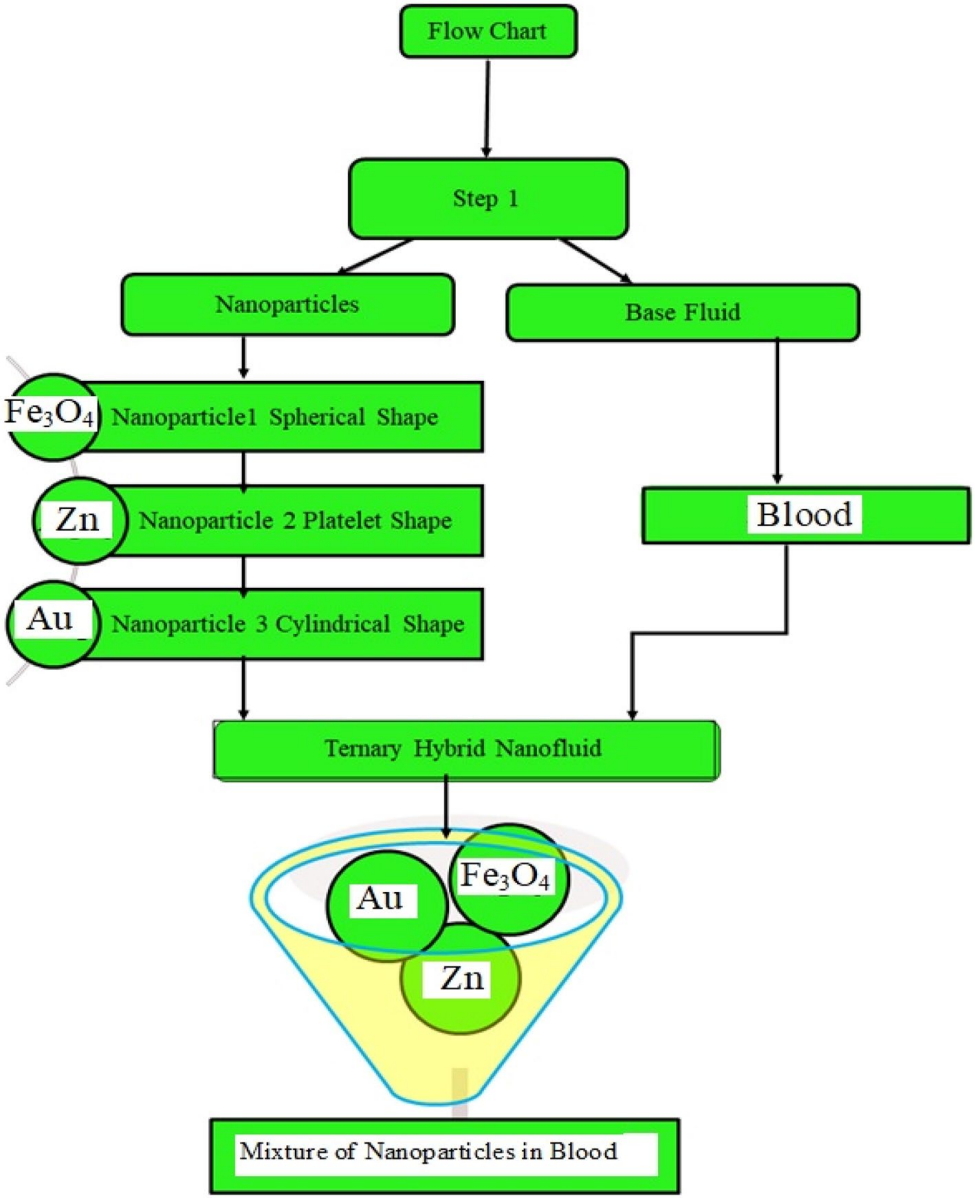
The preparation method for ternary hybrid nanofluid.

The present research highlights the impact of fluid glow in channel with one plate is stationary and the second one is moving in the presence of the external pressure in fluid direction. This kind of fluid flow in fluid dynamics is known generalized Couette flow. Moreover, we have taken Casson fluid model with the addition of couple stresses between the two plates. The base fluid is blood which is filled up by three different shaped nanoparticles for highlighting its impact in the heat transfer rate. This flow is time dependent and laminar flow along with the pressure exerted on the fluid in channel. In addition to this the lower plate is disturb with constant velocity and the upper plate is keep stationary. The lower plate temperature is $T_{w}$ and the upper plate has an ambient temperature $T_{h}$. In the given work $(\text{Fe}_{3} \text{O}_{4} + \text{Zn} + \text{Au})/{\text{blood}}$ based ternary hybrid nanofluids is considered. Furthermore, we focus on the analysis of spherical shaped $\text{Fe}_{3} \text{O}_{4}$, platelet shaped $\left( \text{Zn} \right)$, and cylindrical shaped gold $\left( \text{Au} \right)$ within the base fluid blood. Additionally, the couple stress Casson ternary hybrid nanofluid moving in $x$-direction due to the constant pressure gradient $G$ as shown in Fig. 1 which illustrates the problem geometry. Moreover, the flow chart of the of the Tri-hybrid nanofluid is shown in Fig. 2.

In the view of assumptions considered in this study, the governing equations along with IC’s and BC’s which are explained in^2,54^. Therefore, we have:

1 $$ \rho_{hnf} \frac{\partial u(y,t)}{{\partial t}} = G^{*} + \mu_{hnf} \left( {1 + \frac{1}{\beta }} \right)\frac{{\partial^{2} u(y,t)}}{{\partial y^{2} }} - \eta \frac{{\partial^{4} u(y,t)}}{{\partial y^{4} }} + g\left( {\rho \beta_{T} } \right)_{hnf} \left( {T - T_{\infty } } \right), $$

2 $$ \left( {\rho C_{p} } \right)_{hnf} \frac{\partial T(y,t)}{{\partial t}} = k_{hnf} \frac{{\partial^{2} y(y,t)}}{{\partial y^{2} }}, $$

3 $$\left. {\begin{array}{*{20}ll}    u(y,0) = 0,\,\,\,T(y,0) = 0, & {\text{for}}\,\,\,0 \le y \le h  \\    u(0,t) = H\left( t \right)U_{0} ,\,\,T(0,t) = T_{w} , & { {\text{for}}\,\,t > 0,}  \\    u(h,t) = 0,\,\,T(h,t) = T_{h} , & {\text{for}}\,\,t > 0,  \\ \frac{{\partial^{2} u(0,t)}}{{\partial y^{2} }} = \frac{{\partial^{2} u(h,t)}}{{\partial y^{2} }} = 0, & {\text{for}}\,\,t > 0.  \\   \end{array} } \right\}.$$

where $u\,\,{\text{and}}\,\,T$ represents velocity and temperature, $\beta \,\,{\text{and}}\,\,\eta$ represents Casson parameter and couple stress parameter respectively. Similarly, $\rho_{hnf} \,\,{\text{and}}\,\,\mu_{hnf}$ represents density and viscosity of tri-hybrid nanofluid respectively. Finally,$\,\left( {\rho C_{p} } \right)_{hnf} {,}\left( {\rho \beta_{T} } \right)_{hnf} \,{\text{and}}\,\,k_{hnf}$ represents heat capacitance, coefficient of thermal conductivity and thermal conductivity of tri-hybrid nanofluid.

## The mathematical expressions for the tri-hybrid nanofluid

This section provide a brief explanation of the heat properties of tri-hybrid nanofluid. In the analysis it has been discussed that these properties of nanoparticles concentration dependent of particles in the base fluid and thermal properties of the working base fluid. The properties of the considered nanoparticles and base fluid are provided in Table 1. Furthermore, the impact of shape factor e.g., platelet, spherical and cylindrical type nanoparticles are considered in blood. The tri-hybrid nano-liquid can be evaluated using the idea given in^36,41,42^, hence having:

4 $$ \rho_{hnf} = \left( {1 - \phi_{1} - \phi_{2} - \phi_{3} } \right)\rho_{bf} + \phi_{1} \rho_{sp1} + \phi_{2} \rho_{sp2} + \phi_{3} \rho_{sp3} , $$

5 $$ \left( {\rho C_{p} } \right)_{hnf} = \left( {1 - \phi_{1} - \phi_{2} - \phi_{3} } \right)\left( {\rho C_{p} } \right)_{bf} + \phi_{1} \left( {\rho C_{p} } \right)_{sp1} + \phi_{2} \left( {\rho C_{p} } \right)_{sp2} + \phi_{3} \left( {\rho C_{p} } \right)_{sp3} , \, $$

6 $$ \left( {\rho \beta_{T} } \right)_{hnf} = \left( {1 - \phi_{1} - \phi_{2} - \phi_{3} } \right)\left( {\rho \beta_{T} } \right)_{bf} + \phi_{1} \left( {\rho \beta_{T} } \right)_{sp1} + \phi_{2} \left( {\rho \beta_{T} } \right)_{sp2} + \phi_{3} \left( {\rho \beta_{T} } \right)_{sp3} , \, $$

**Table 1:** Heat characteristics of the base fluid and nanoparticles^55–58^.

Parameters	Blood	$\text{Fe}_{3} \text{O}_{4}$	Zn	Au
$\rho \; \left( {\text{kg}/\text{m}^{3} } \right)$	1053	5200	7140	19,300
$k \; \left( {\text{W}}/\text{m K} \right)$	0.492	6	116	318
$C_{p} \;\left( {\text{J}}/\text{kg K} \right)$	3594	670	389	129
$\beta \;\left( {1/\text{K}} \right)$	0.8	1.3	3.5	1.41
Shape	–	Spherical	Platelet	Cylindrical

Let us underline that the concentration of suspended particles in the base fluid is unable to highlight the thermal properties like, viscosity and thermal conductivity of the tri-hybrid nano-liquid, since we also have a dependence from the shape of the nanoparticles. This is the reason that why, we take the mixture of three different size and shaped of nanoparticles in the base fluid blood, as tri-hybrid nano-liquid leading us also to provide a theoretical analysis of the impact of the concentration and shape factor of nanoparticles.

The characteristics properties of these nanoparticles for tri-hybrid nano-liquid can be expressed as defined by Maxwell^59^, leading to:

7 $$ \frac{{k_{nf1} }}{{k_{bf} }} = \frac{{k_{1} + (n - 1)k_{bf} + (n - 1)\phi (k_{1} - k_{bf} )}}{{k_{1} + (n - 1)k_{bf} - (k_{1} - k_{bf} )}} $$

where $n$ indicates the shape factor $n = \left( {\frac{3}{\Psi }} \right)$, while $\Psi$ represents the spherical ratio.

When we take $\Psi = 1$ and $n = 1$ make them spherical shape of nanoparticles.

By taking $\Psi = 0.612$ and $n = 4.9$ in the Maxwell formula defined above, then the nanoparticles shape become cylindrical.

Similarly by considering the values of $\Psi = 0.52$ and $n = 5.7$ we get the platelet shape of nanoparticles.

Let us underline the expressions for viscosity and thermal conductivity of nanoparticles have different shaped e.g., cylindrical, spherical and platelet shape for tri-hybrid nano-liquid defined by mixture model^60^, leading to:

For spherical shaped of nanoparticles the expression is given by:

8 $$ \left. \begin{aligned} & \frac{{\mu_{nf1} }}{{\mu_{bf} }} = 1 + 2.5\phi_{hnf} + 6.2\phi_{hnf}^{2} \hfill \\ & \frac{{k_{nf1} }}{{k_{bf} }} = \frac{{k_{1} + 2k_{bf} + 2\phi_{hnf} (k_{1} - k_{bf} )}}{{k_{1} + 2k_{bf} - \phi_{hnf} (k_{1} - k_{bf} )}} \hfill \\ \end{aligned} \right\} \mapsto \;\left( {{\text{Spherical nanoparticle-}}{1}} \right), $$

For cylindrical shaped of nanoparticles the expression becomes:

9 $$ \left. \begin{aligned} & \frac{{\mu_{nf2} }}{{\mu_{bf} }} = 1 + 13.5\phi_{hnf} + 904.4\phi_{hnf}^{2} \hfill \\ & \frac{{k_{nf2} }}{{k_{bf} }} = \frac{{k_{2} + 3.9k_{bf} + 3.9\phi_{hnf} (k_{2} - k_{bf} )}}{{k_{2} + 3.9k_{bf} - \phi_{hnf} (k_{2} - k_{bf} )}} \hfill \\ \end{aligned} \right\} \mapsto \;\left( {{\text{Cylindrical nanoparticle-}}{2}} \right), $$

For the platelet type of nanoparticles the expression becomes:

10 $$ \left. \begin{aligned} & \frac{{\mu_{nf3} }}{{\mu_{bf} }} = 1 + 37.1\phi_{hnf} + 612.6\phi_{hnf}^{2} \hfill \\ & \frac{{k_{nf3} }}{{k_{bf} }} = \frac{{k_{3} + 4.7k_{bf} + 4.7\phi_{hnf} (k_{3} - k_{bf} )}}{{k_{3} + 4.7k_{bf} - \phi_{hnf} (k_{3} - k_{bf} )}} \hfill \\ \end{aligned} \right\} \mapsto \;\left( {{\text{Platelet nanoparticle-}}{3}} \right) $$

Since, this study is focused on the applications of tri-hybrid nano-liquids with the dispersion of three different nanoparticles of the type e.g., platelet, spherical and cylindrical. The tri-hybrid nano-liquid is created by the addition of the above mentioned nanoparticles in the blood. For the effectiveness of the present model nanofluid expressions have been obtained by considering the method of interpolation which is concentration dependent and shape factor of the nanoparticles 1, 2 and 3 which is represented by $\phi_{1} ,\,\,\phi_{2} \,\,{\text{and}}\,\,\phi_{3}$ respectively.

The corresponding expression for the viscosity of tri-hybrid nano-liquid can be written as:

11 $$ \mu_{hnf} = \frac{{\mu_{nf1} \phi_{1} + \mu_{nf2} \phi_{2} + \mu_{nf3} \phi_{3} }}{{\phi_{hnf} }} $$

The expression of the thermal conductivity of tri-hybrid nano-liquid can be written as:

12 $$ k_{hnf} = \frac{{k_{nf1} \phi_{1} + k_{nf2} \phi_{2} + k_{nf3} \phi_{3} }}{{\phi_{hnf} }} $$

Here it is worth noting that the collective concentrations of all the three different nanoparticles gave the concentration of tri-hybrid nano-liquids which can be written as $\phi_{hnf} = \phi_{1} + \phi_{2} + \phi_{3}$. For better understanding of the readers here it is assumed that $\phi_{1}$ represents spherical $\left( {\text{Fe}_{3} \text{O}_{4} } \right)$ nanoparticles, $\phi_{2}$ represents platelet shaped $\left( \text{Zn} \right)$ nanoparticles and $\phi_{3}$ represents cylindrical gold $(\text{Au})$ nanoparticles.

The dimensionless quantities considered for dimensional analysis are given below, with respect to Eqs. (1–3), hence obtaining:

$$ \xi = \frac{y}{h};\,\,\,\,\,\,w = \frac{u}{{U_{0} }};\,\,\,\,\,\,\,\,\tau = \frac{{U_{0} t}}{h},\,\,\,\,\,\theta = \frac{{T - T_{h} }}{{T_{w} - T_{h} }}. $$

13 $$ B_{0} \frac{\partial w(\xi ,\tau )}{{\partial \tau }} = G + \frac{{\partial^{2} w(\xi ,\tau )}}{{\partial \xi^{2} }} - \lambda \frac{{\partial^{4} w(\xi ,\tau )}}{{\partial \xi^{4} }} + B_{2} \theta \left( {\xi ,\tau } \right), $$

14 $$ B_{3} \frac{\partial \theta (y,\tau )}{{\partial \tau }} = \frac{{\partial^{2} \theta (\xi ,\tau )}}{{\partial \xi^{2} }}, $$

15 $$\left. {\begin{array}{*{20}ll}    w = 0,\theta = 0, & \quad  {\text{for}}\,\,\,0 \le \xi \le 1\,\,{\text{and}}\,\tau = 0,  \\    w = 1,\theta = 1, & \quad {\text{for}}\,\,\,\xi = 0\,\,\,\,{\text{and}}\,\,\,\,\tau > 0,  \\    w = 0,\theta = 0, & \quad {\text{for}}\,\,\,\xi = 1\,\,\,\,{\text{and}}\,\,\,\,\tau > 0,  \\  \frac{{\partial^{2} w}}{{\partial \xi^{2} }} = 0, & \quad {\text{at}}\,\,\,\xi = 0\,\,\,\,{\text{and }}\xi = 1.  \\   \end{array} } \right\}.$$

where

$$ \chi_{1} = \left( {1 - \phi_{1} - \phi_{2} - \phi_{3} } \right) + \frac{{\phi_{1} \rho_{sp1} }}{{\rho_{bf} }} + \frac{{\phi_{2} \rho_{sp2} }}{{\rho_{bf} }} + \frac{{\phi_{3} \rho_{sp3} }}{{\rho_{bf} }},\,\,\chi_{2} = 1 + 2.5\phi + 6.2\phi^{2} ,\,\,\, $$

$$ \,\chi_{3} = 1 + 13.5\phi + 904.4\phi^{2} ,\,\,\,\,\chi_{4} = 1 + 37.1\phi + 612.6\phi^{2} ,\,\,\,\chi_{5} = \frac{{\chi_{2} \phi_{1} + \chi_{3} \phi_{2} + \chi_{4} \phi_{3} }}{{\phi_{hnf} }}, $$

$$ \chi_{6} = \left( {1 - \phi_{1} - \phi_{2} - \phi_{3} } \right) + \frac{{\phi_{1} \left( {\rho \beta_{T} } \right)_{sp1} }}{{\left( {\rho \beta_{T} } \right)_{bf} }} + \frac{{\phi_{2} \left( {\rho \beta_{T} } \right)_{sp2} }}{{\left( {\rho \beta_{T} } \right)_{bf} }} + \frac{{\phi_{3} \left( {\rho \beta_{T} } \right)_{sp3} }}{{\left( {\rho \beta_{T} } \right)_{bf} }}, \, $$

$$ \chi_{7} = \left( {1 - \phi_{1} - \phi_{2} - \phi_{3} } \right) + \frac{{\phi_{1} \left( {\rho C_{p} } \right)_{sp1} }}{{\left( {\rho C_{p} } \right)_{bf} }} + \frac{{\phi_{2} \left( {\rho C_{p} } \right)_{sp2} }}{{\left( {\rho C_{p} } \right)_{bf} }} + \frac{{\phi_{3} \left( {\rho C_{p} } \right)_{sp3} }}{{\left( {\rho C_{p} } \right)_{bf} }}, $$

$$ \,\,\chi_{8} = \frac{{k_{1} + 2k_{bf} + 2\phi (k_{1} - k_{bf} )}}{{k_{1} + 2k_{bf} - (k_{1} - k_{bf} )}},\,\,\chi_{9} = \frac{{k_{2} + 3.9k_{bf} + 3.9\phi (k_{2} - k_{bf} )}}{{k_{2} + 3.9k_{bf} - (k_{2} - k_{bf} )}},\,\,\,\,\,\, $$

$$ \chi_{10} = \frac{{k_{3} + 4.7k_{bf} + 4.7\phi (k_{3} - k_{bf} )}}{{k_{3} + 4.7k_{bf} - (k_{3} - k_{bf} )}},\,\,\chi_{11} = \frac{{\chi_{8} \phi_{1} + \chi_{9} \phi_{2} + \chi_{10} \phi_{3} }}{{\phi_{hnf} }} $$

$$ B_{0} = \frac{{Re.\chi_{1} }}{{\beta .\chi_{5} }},\,\,B_{1} = \frac{{\chi_{6} }}{{\chi_{5} }},\,\,B_{2} = \frac{{B_{1} .Gr}}{\beta },\,\,B_{3} = \frac{{Pr.\chi_{7} .Re}}{{\chi_{11} }} $$

$$ \,Gr = \frac{{g\beta_{T} \left( {T_{w} - T_{0} } \right)h^{2} }}{{\upsilon U_{0} }},\,\,Pr = \frac{{\mu C_{p} }}{k}\,,\,\,\,Re = \frac{{U_{0} h}}{\upsilon },\,\,\lambda = \frac{\eta }{{\chi_{5} \beta \mu h^{2} }},\,\,G = \frac{{G^{*} h^{2} }}{{U_{0} \chi_{5} \beta \mu }}. $$

Let us remark that $Re,\,Pr,\,Gr$ represents Reynolds, Prandtl and Grashof number respectively. Similarly $G$ represents the pressure, $\beta$ represents Casson parameter and $\lambda$ represents the couple stress parameter.

## Solutions of the problem with AB-fractional derivatives

Incorporating AB-fractional operator on the fluid model stated before, we get the generalized fractional model given below:

16 $$^{AB} {\mathbf{D}}_{\tau }^{\alpha } B_{0} w(\xi ,\tau ) = G + \frac{{\partial^{2} w(\xi ,\tau )}}{{\partial \xi^{2} }} - \lambda \frac{{\partial^{4} w(\xi ,\tau )}}{{\partial \xi^{4} }} + B_{2} \theta \left( {\xi ,\tau } \right), $$

17 $$^{AB} {\mathbf{D}}_{\tau }^{\alpha } B_{3} \theta (\xi ,\tau ) = \frac{{\partial^{2} \theta (\xi ,\tau )}}{{\partial \xi^{2} }}. $$

Here $^{AB} {\mathbf{D}}_{\tau }^{\alpha } (.)$ express the AB-fractional operator defined^61^.

18 $$^{AB} {\mathbf{D}}_{\tau }^{\beta } (\tau ) = \frac{N\left( \beta \right)}{{\left( {1 - \beta } \right)}}\int\limits_{0}^{\tau } {E_{\beta } \left( {\frac{{ - \beta \left( {\tau - t} \right)^{\beta } }}{1 - \beta }} \right)} f^{^{\prime}} \left( \tau \right)dt, $$

where $N\left( \beta \right)$ express the normalization function, which gave us the result as $N\left( 1 \right) = N\left( 0 \right) = 1$ and $\beta \in \left( {0,1} \right).$

Mittag–Leffler function can be expressed as^62^.

19 $$ E_{\beta } \left( { - t^{\beta } } \right) = \sum\limits_{k = 0}^{\infty } {\frac{{\left( { - t} \right)^{\beta k} }}{{\Gamma \left( {\beta k + 1} \right)}}.} $$

### Solutions of temperature for AB-fractional operator

As to obtain the closed form solutions of the stated problem of aforementioned PDEs, first consider the applications of the Laplace to Eq. (17) imposing the IC’s stated in Eq. (15), so that the transform solution obtained as:

20 $$ \frac{{q^{\alpha } B_{3} \overline{\theta } \left( {\xi ,q} \right)}}{{\left( {1 - \alpha } \right)q^{\alpha } + \alpha }} = \frac{{d^{2} \overline{\theta } \left( {\xi ,q} \right)}}{{d\xi^{2} }}, $$

21 $$ \frac{{r_{1} .q^{\alpha } .\overline{\theta } \left( {\xi ,q} \right)}}{{q^{\alpha } + r_{2} }} = \frac{{d^{2} \overline{\theta } \left( {\xi ,q} \right)}}{{d\xi^{2} }}, $$

Now apply sine finite Fourier transform to Eq. (21), then obtaining:

22 $$ \overline{\theta }_{F} \left( {n,q} \right) = \left( {\frac{{r_{5} \left( {q^{\alpha } + r_{2} } \right)}}{{q\left( {q^{\alpha } + r_{4} } \right)}}} \right). $$

Equation (22), can be more conveniently rewritten as:

23 $$ \overline{\theta }_{F} \left( {n,q} \right) = \frac{{r_{5} .r_{2} }}{{r_{4} .q}} + \frac{{r_{5} \left( {r_{4} - r_{2} } \right)}}{{r_{4} }}\frac{1}{{q^{1 - \alpha } \left( {q^{\alpha } + r_{4} } \right)}}. $$

with inverse Laplace that reads as:

24 $$ \overline{\theta }_{F} \left( {n,\tau } \right) = \frac{{r_{5} .r_{2} }}{{r_{4} }} + \frac{{r_{5} \left( {r_{4} - r_{2} } \right)}}{{r_{4} }}h\left( t \right) * F_{\beta } \left( { - r_{4} ,\tau } \right). $$

25 $$ \overline{\theta }_{F} \left( {n,\tau } \right) = \frac{1}{{\sigma_{n} }} + \frac{{r_{5} \left( {r_{4} - r_{2} } \right)}}{{r_{4} }}h\left( t \right) * F_{\beta } \left( { - r_{4} ,\tau } \right). $$

In order to get the solutions in original domain inverting the Fourier transform to above equation obtaining the following results, see^63,64^:

26 $$ \theta \left( {\xi ,\tau } \right) = 1 - \frac{\xi }{h} - \left( {\frac{\xi (h - 1)}{h}} \right) + \frac{2}{h}\sum\limits_{n = 1}^{\infty } {\frac{{r_{5} \left( {r_{4} - r_{2} } \right)}}{{r_{4} }}h\left( t \right) * F_{\beta } \left( { - r_{4} ,\tau } \right)} \sin \left( {\frac{n\pi \xi }{h}} \right). $$

### Solutions of momentum equation for AB-fractional operator

By applying the Laplace transform to Eq. (16) and exploiting the IC’s from Eq. (15), the following solutions are obtained:

27 $$ \frac{{B_{0} \,q^{\alpha } }}{{\left( {1 - \alpha } \right)q^{\alpha } + \alpha }}\overline{w} (\xi ,q) = \frac{G}{q} + \frac{{d^{2} \overline{w} (\xi ,q)}}{{d\xi^{2} }} - \lambda \frac{{d^{4} \overline{w} (\xi ,q)}}{{d\xi^{4} }} + B_{2} \overline{\theta } \left( {\xi ,q} \right). $$

Using the applications of Fourier transform to Eq. (27) obtaining the following results:

28 $$ \frac{{r_{6} \,\,q^{\alpha } }}{{q^{\alpha } + r_{2} }}\overline{w}_{F} (n,q) = \frac{{G\left( {1 - \left( { - 1} \right)^{n} } \right)}}{{q\sigma_{n} }} + \frac{{\sigma_{n} }}{p} - \sigma_{n}^{2} \overline{w}_{F} (n,q) + \lambda \frac{{\sigma_{n}^{3} }}{q} - \lambda \sigma_{n}^{4} \overline{w}_{F} (n,q) + B_{2} \overline{\theta }_{F} \left( {n,q} \right). $$

29 $$ \overline{w}_{F} (n,q) = \left( {\frac{{G\left( {1 - \left( { - 1} \right)^{n} } \right) + \sigma_{n}^{2} + \lambda \sigma_{n}^{4} }}{{q\sigma_{n} }}} \right) \times \left( {\frac{{q^{\alpha } + r_{2} }}{{r_{7} \left( {q^{\alpha } + r_{8} } \right)}}} \right) + B_{2} \overline{\theta }_{F} \left( {n,q} \right) \times \left( {\frac{{q^{\alpha } + r_{2} }}{{r_{7} \left( {q^{\alpha } + r_{8} } \right)}}} \right). $$

Substituting the results of $\overline{\theta }_{F} \left( {n,q} \right)$ given in Eq. (22) into the above Eq. (29), obtaining

30 $$ \overline{w}_{F} (n,q) = \left( {\frac{{G\left( {1 - \left( { - 1} \right)^{n} } \right) + \sigma_{n}^{2} + \lambda \sigma_{n}^{4} }}{{r_{7} \sigma_{n} }}} \right) \times \left( {\frac{{q^{\alpha } + r_{2} }}{{q\left( {q^{\alpha } + r_{8} } \right)}}} \right) + \frac{{B_{2} r_{5} }}{{r_{7} }}\left( {\frac{{\left( {q^{\alpha } + r_{2} } \right)}}{{q\left( {q^{\alpha } + r_{4} } \right)}}} \right). \times \left( {\frac{{q^{\alpha } + r_{2} }}{{\left( {q^{\alpha } + r_{8} } \right)}}} \right). $$

To write in more convenient way applying partial fraction to RHS of Eq. (30) obtaining:

31 $$ \begin{aligned} \overline{w}_{F} (n,q) & = \left( {\frac{{G\left( {1 - \left( { - 1} \right)^{n} } \right) + \sigma_{n}^{2} + \lambda \sigma_{n}^{4} }}{{r_{7} \sigma_{n} }}} \right) \times \left( {\frac{{r_{2} }}{{r_{8} \,q}} + \frac{{\left( {r_{8} - r_{2} } \right)}}{{r_{8} \,q^{1 - \alpha } \left( {q^{\alpha } + r_{8} } \right)}}} \right) \hfill \\ & \quad + \frac{{B_{2} r_{5} }}{{r_{7} }}\left[ {\frac{1}{q} - \frac{{\left( {r_{2} - r_{4} } \right)^{2} }}{{\left( {r_{4} - r_{8} } \right)q\left( {q^{\alpha } + r_{4} } \right)}} + \frac{{\left( {r_{2} - r_{8} } \right)^{2} }}{{\left( {r_{4} - r_{8} } \right)q\left( {q^{\alpha } + r_{8} } \right)}}} \right]. \hfill \\ \end{aligned} $$

By inverting the Laplace transform obtaining the following result:

32 $$ \begin{aligned} w_{F} (n,\tau ) & = \left( {\frac{{G\left( {1 - \left( { - 1} \right)^{n} } \right) + \sigma_{n}^{2} + \lambda \sigma_{n}^{4} }}{{\sigma_{n} }}} \right) \times \left( {\frac{{r_{2} }}{{r_{7} \,r_{8} \,}} + \frac{{\left( {r_{8} - r_{2} } \right)}}{{r_{7} \,r_{8} \,}}h(t) * F_{\beta } \left( { - r_{8} ,\tau } \right)} \right) \hfill \\ & \quad + \frac{{B_{2} r_{5} }}{{r_{7} }}\left[ {1 - \frac{{\left( {r_{2} - r_{4} } \right)^{2} }}{{\left( {r_{4} - r_{8} } \right)}}1 * F_{\beta } \left( { - r_{4} ,\tau } \right) + \frac{{\left( {r_{2} - r_{8} } \right)^{2} }}{{\left( {r_{4} - r_{8} } \right)}}1 * F_{\beta } \left( { - r_{8} ,\tau } \right)} \right]. \hfill \\ \end{aligned} $$

where

33 $$ L^{ - 1} \left( {\frac{1}{{q^{1 - \beta } }}} \right) = h(t) = \frac{1}{{t^{\beta } \Gamma (1 - \beta )}}\,, $$

34 $$ F_{\beta } \left( { - r_{i} ,\tau } \right) = L^{ - 1} \left( {\frac{1}{{q^{\beta } + r_{i} }}} \right)\, = \sum\limits_{n = 0}^{\infty } {\frac{{\left( { - r_{i} } \right)^{n} \tau^{(n + 1)\beta - 1} }}{{\Gamma \left( {(n + 1)\beta } \right)}}\,,\,\,} {\text{where}}\,\,r_{i} = r_{4} \;{\text{and}}\,\,r_{8} . $$

From the above results $F_{\beta } (.,.)$ represents a function which is introduce by Robotnov and Hartleys'^65^.

Equation (32) can be re-written more conveniently as:

35 $$ \begin{aligned} w_{F} (n,\tau ) & = \left( {\frac{{G\left( {1 - \left( { - 1} \right)^{n} } \right) + \sigma_{n}^{2} + \lambda \sigma_{n}^{4} }}{{\sigma_{n} \left( {\sigma_{n}^{2} + \lambda \sigma_{n}^{4} } \right)}}} \right) + \left( {\frac{{G\left( {1 - \left( { - 1} \right)^{n} } \right) + \sigma_{n}^{2} + \lambda \sigma_{n}^{4} }}{{\sigma_{n} }}} \right) \times \left( {\frac{{\left( {r_{8} - r_{2} } \right)}}{{r_{7} \,r_{8} \,}}h(t) * F_{\beta } \left( { - r_{8} ,\tau } \right)} \right) \hfill \\  & \quad + \left[ {\left( {\frac{{B_{2} r_{5} }}{{r_{7} }}} \right) - \left( {\frac{{B_{2} r_{5} }}{{r_{7} }}} \right)\frac{{\left( {r_{2} - r_{4} } \right)^{2} }}{{\left( {r_{4} - r_{8} } \right)}}1 * F_{\beta } \left( { - r_{4} ,\tau } \right) + \left( {\frac{{B_{2} r_{5} }}{{r_{7} }}} \right)\frac{{\left( {r_{2} - r_{8} } \right)^{2} }}{{\left( {r_{4} - r_{8} } \right)}}1 * F_{\beta } \left( { - r_{8} ,\tau } \right)} \right], \hfill \\ \end{aligned} $$

36 $$ \begin{aligned} w_{F} (n,\tau ) & = \left( {\frac{{G\left( {1 - \left( { - 1} \right)^{n} } \right) + \sigma_{n}^{2} + \lambda \sigma_{n}^{4} }}{{\sigma_{n} \left( {\sigma_{n}^{2} + \lambda \sigma_{n}^{4} } \right)}}} \right) + A_{1} \left( {h(t) * F_{\beta } \left( { - r_{8} ,\tau } \right)} \right) \hfill \\ & \quad + \left[ {A_{2} - A_{3} \left( {1 * F_{\beta } \left( { - r_{4} ,\tau } \right)} \right) + A_{4} \left( {1 * F_{\beta } \left( { - r_{8} ,\tau } \right)} \right)} \right]. \hfill \\ \end{aligned} $$

and we can rewrite Eq. (36) as follows:

37 $$ \begin{aligned} w_{s} (n,\tau ) & = \left( {\frac{{\left( {1 - \left( { - 1} \right)^{n} } \right)}}{{\sigma_{n} }} + \frac{{\left( { - 1} \right)^{n} }}{{\sigma_{n} }} - \frac{{G\left( {1 - \left( { - 1} \right)^{n} } \right)}}{{\sigma_{n} }} + \frac{{G\left( {1 - \left( { - 1} \right)^{n} } \right)}}{{\sigma_{n}^{3} }} + \frac{{G\left( {1 - \left( { - 1} \right)^{n} } \right)}}{{\left( {1 + \sigma_{n}^{2} } \right)}}} \right) \hfill \\ & \quad + \left( {A_{1} h(t) * F_{\beta } \left( { - r_{8} ,\tau } \right)} \right) + \left[ {A_{2} - A_{3} \left( {1 * F_{\beta } \left( { - r_{4} ,\tau } \right)} \right) + A_{4} \left( {1 * F_{\beta } \left( { - r_{8} ,\tau } \right)} \right)} \right]. \hfill \\ \end{aligned} $$

as to then apply the inverse sine-Fourier transform to obtain , see^63,64^, the following form:

38 $$ \begin{aligned} w(\xi ,\tau ) &= \left( {1 - G - \left( {\frac{1}{h} - \frac{Gh}{2}} \right)\xi - \frac{G}{2}\xi^{2} + G\left\{ {\frac{{\cosh \left( {\frac{h}{2} - \xi } \right)}}{{\cosh \left( \frac{h}{2} \right)}}} \right\}} \right) \hfill \\ & \quad + \frac{2}{h}\sum\limits_{n = 1}^{\infty } {\left[ {A_{1} h(t) * F_{\beta } \left( { - r_{8} ,\tau } \right)} \right]} \sin \left( {\sigma_{n} \xi } \right) \hfill \\ & \quad + \frac{2}{h}\sum\limits_{n = 1}^{\infty } {\left[ {A_{2} - A_{3} \left( {1 * F_{\beta } \left( { - r_{4} ,\tau } \right)} \right) + A_{4} \left( {1 * F_{\beta } \left( { - r_{8} ,\tau } \right)} \right)} \right]} \sin \left( {\sigma_{n} \xi } \right), \hfill \\ \end{aligned} $$

The final solutions obtained above are the sum of steady and unsteady solutions.

From above result the study solution can be expressed as:

39 $$ w_{p} \left( \xi \right) = 1 - G - \left( {\frac{1}{h} - \frac{Gh}{2}} \right)\xi - \frac{G}{2}\xi^{2} + G\left\{ {\frac{{\cosh \left( {\frac{h}{2} - \xi } \right)}}{{\cosh \left( \frac{h}{2} \right)}}} \right\}, $$

and the unsteady part of the solution is given below:

40 $$ w_{\tau } \left( {\xi ,\tau } \right) = \frac{2}{h}\sum\limits_{n = 1}^{\infty } {\left[ {A_{1} h(t) * F_{\beta } \left( { - r_{8} ,\tau } \right)} \right]} \sin \left( {\sigma_{n} \xi } \right) + \frac{2}{h}\sum\limits_{n = 1}^{\infty } {\left[ \begin{gathered} A_{2} - A_{3} \left( {1 * F_{\beta } \left( { - r_{4} ,\tau } \right)} \right) \hfill \\ + A_{4} \left( {1 * F_{\beta } \left( { - r_{8} ,\tau } \right)} \right) \hfill \\ \end{gathered} \right]} \sin \left( {\sigma_{n} \xi } \right). $$

where

$$ r_{1} = \frac{{B_{3} }}{1 - \alpha },\,\,r_{2} = \frac{\alpha }{1 - \alpha },\,\,\sigma_{n} = \frac{n\pi }{h},\,\,r_{3} = r_{1} + \sigma_{n}^{2} ,\,\,r_{4} = \frac{{r_{2} \,\sigma_{n}^{2} }}{{r_{1} + \sigma_{n}^{2} }},\,\,r_{5} = \frac{{\sigma_{n} }}{{r_{3} }} $$

$$ r_{6} = \frac{{B_{0} }}{1 - \alpha },\,\,\,r_{7} = r_{6} + \sigma_{n}^{2} + \lambda \sigma_{n}^{4} ,\,\,\,r_{8} = \frac{{r_{2} \sigma_{n}^{2} + \lambda r_{2} \sigma_{n}^{4} }}{{r_{6} + \sigma_{n}^{2} + \lambda \sigma_{n}^{4} }}. $$

$$ A_{1} = \left( {\frac{{G\left( {1 - \left( { - 1} \right)^{n} } \right) + \sigma_{n}^{2} + \lambda \sigma_{n}^{4} }}{{\sigma_{n} }}} \right)\frac{{\left( {r_{8} - r_{2} } \right)}}{{r_{7} \,r_{8} \,}},\,\,\,A_{2} = \left( {\frac{{B_{2} r_{5} }}{{r_{7} }}} \right),\, $$

$$ \,A_{3} = \left( {\frac{{B_{2} r_{5} }}{{r_{7} }}} \right)\frac{{\left( {r_{2} - r_{4} } \right)^{2} }}{{\left( {r_{4} - r_{8} } \right)}},\,\,A_{4} = \left( {\frac{{B_{2} r_{5} }}{{r_{7} }}} \right)\frac{{\left( {r_{2} - r_{8} } \right)^{2} }}{{\left( {r_{4} - r_{8} } \right)}}. $$

## Limiting cases

### Solution in the absence of external pressure

This section provides a limiting case by putting the effect of some involved parameters such as external pressure $\left( {G = 0} \right)$ and the volume fraction of the nanoparticles $\phi_{1} = \phi_{2} = \phi_{3} = 0$ then our obtained solutions reduced to the solutions obtained by Ahmad et al.^54^. This results shows a strong agreement with the already published work which shows the validity of our obtained results. For clear understanding the present solution have been compared with the already published work of Ahmad et al.^54^ using the graphical analysis which is given in figure^16^.

### Solution in the absence of Casson fluid parameter

In this section we inspected the solutions without Casson fluid parameter $\frac{1}{\beta } \to 0$, $\left( {Gr = 0} \right)$ and $\phi_{1} = \phi_{2} = \phi_{3} = 0$ then our obtained solutions reduced to the solution obtained by Arif et al.^52^ which verify our obtained results. For the comparative analysis both the solutions are plotted in figure^17^.

## Results and discussion

The present analysis aims to visualize the physical exploration of all flow parameters on the flow, its impact on tri-hybrid nano-liquids and the applications in biological and medical sciences. In this study different nanoparticles with shape effect in a single base fluid blood have been considered, particularly studying the case of tri-hybrid nano-liquids. In the base fluid blood the mixture of spherical shaped ferric oxide $\text{Fe}_{3} \text{O}_{4}$, platelet shaped zinc $\left( \text{Zn} \right),$ and cylindrical shaped gold $\left( \text{Au} \right)$ nanoparticles are considered for the advance biomedical applications. The platelet shaped $\left( \text{Zn} \right)$ is used in the blood for decreasing viscosity of the blood which has useful applications in COVID-19 infectious disease. In addition zinc nanoparticles use as preventive therapeutic agent and increase the immune response against COVID-19 infectious disease. Similarly, cylindrical shaped gold $\left( \text{Au} \right)$ nanoparticles have useful applications in the cancer therapy which is used in the blood to hit the cancer cells in the body. The ferric oxide $\text{Fe}_{3} \text{O}_{4}$ nanoparticles have many applications in targeted anticancer drug delivery systems.

The present problem is modeled in terms of PDE’s with physical IC’s and BC’s. The couple stress Casson tri-hybrid nano-liquid flow is taken in channel between two parallel plates. The simple classical model is generalized taking AB fractional derivatives. For the closed form solutions of the considered model of couple stress Casson tri-hybrid nano-liquid the integral transforms (Laplace and Fourier) have been used. The results obtained from the present study are highlighted in graphs for the parameters which affect the fluid flow applying the applications of MATHCAD software. The impact of $\alpha$, $Gr$, $Pr$, $\tau$, $\phi$ on the calculation of Nusselt number and skin friction mentioned in tables.

**Figure 3:**
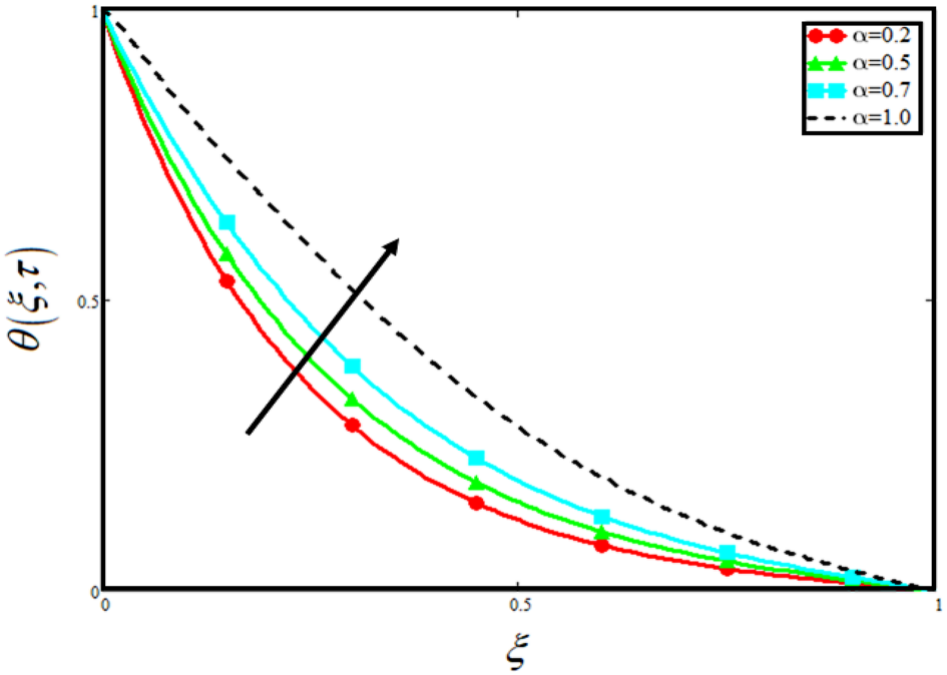
Variation in temperature against different values of $\alpha$ when $\tau = 1$, $\phi_{hnf} = 0.02$, $\Pr = 21$ and ${\text{Re}} = 1.2$.

**Figure 4:**
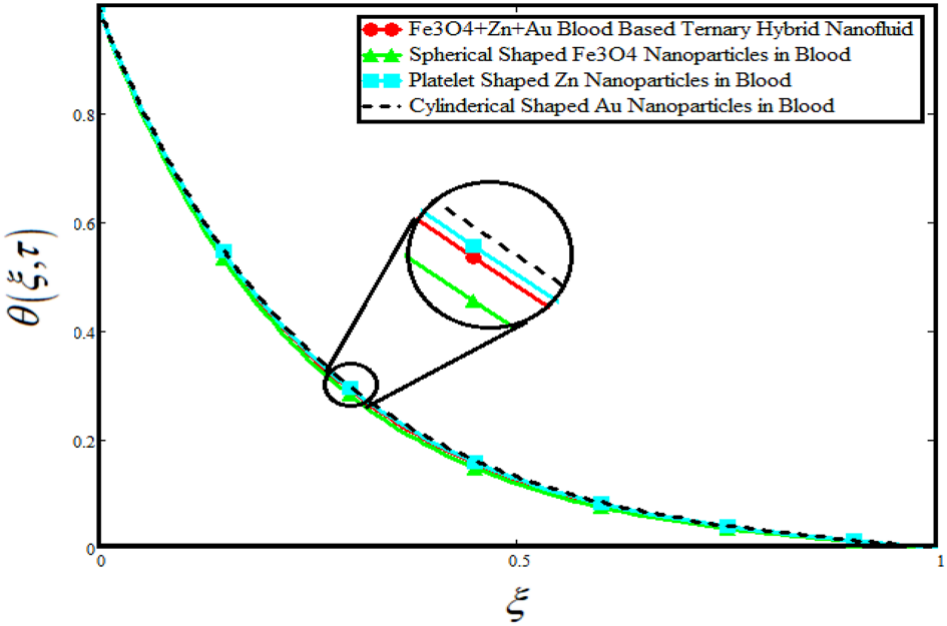
Comparison of temperature distribution with ternary hybrid nanofluids and unitary nanofluid when $\alpha = 0.5$, $\tau = 1$, $\Pr = 21$ and ${\text{Re}} = 1.2$.

**Figure 5:**
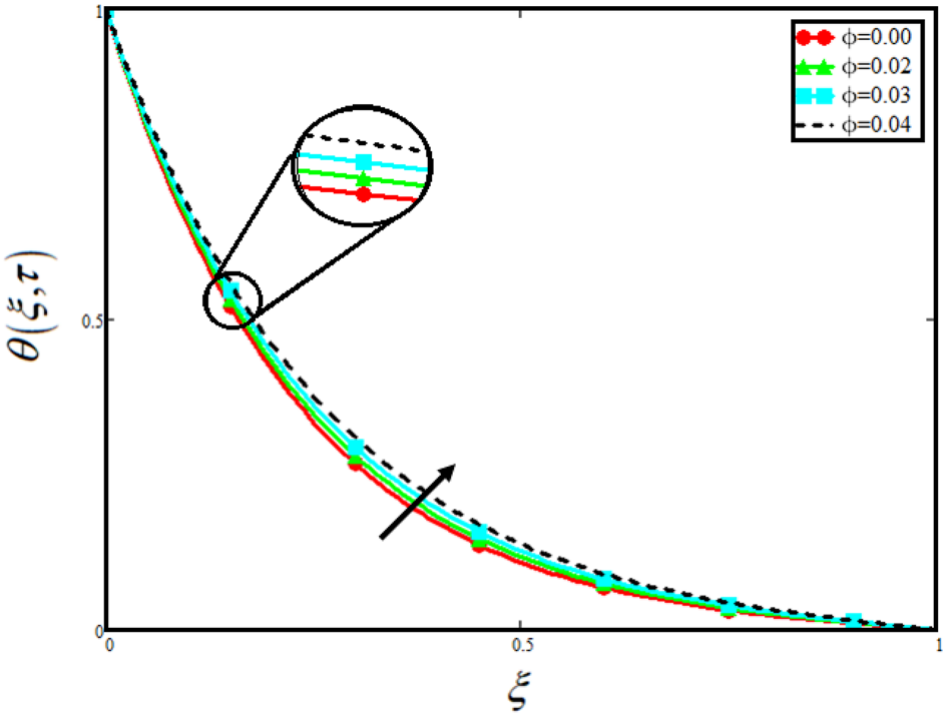
Variation in temperature against different values of $\phi_{hnf}$ when $\tau = 1$, $\alpha = 0.5$, $\Pr = 21$ and ${\text{Re}} = 1.2$.

**Figure 6:**
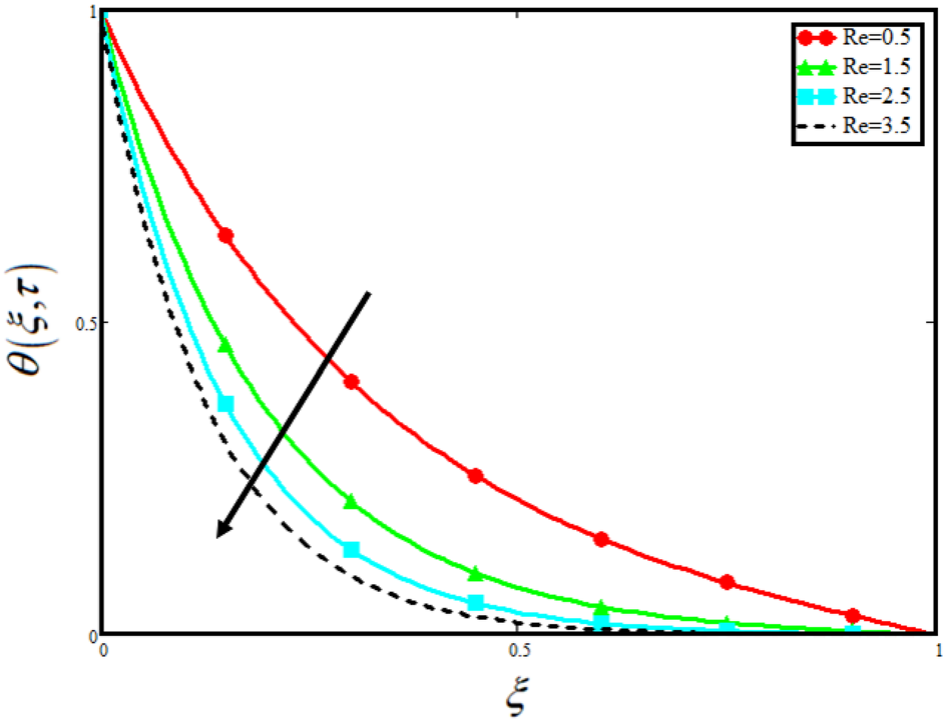
Variation in temperature against different values of ${\text{Re}}$ when $\tau = 1$, $\phi_{hnf} = 0.02$, $\Pr = 21$ and $\alpha = 0.5$.

**Figure 7:**
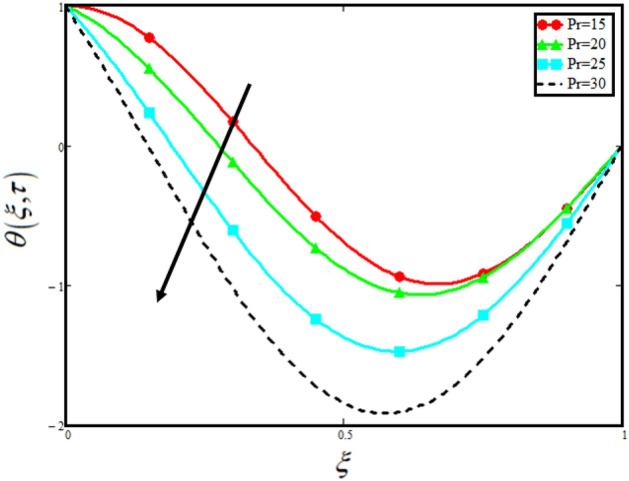
Variation in temperature against different values of $\Pr$ when $\tau = 1$, $\phi_{hnf} = 0.02$, $\alpha = 0.5$ and ${\text{Re}} = 1.2$.

The physical sketch of the problem can be seen in Fig. 1. Figure 2 highlight the flow chart of the tri-hybrid nanofluid the mixture of nanoparticles in the base fluid blood. Figure 3 highlights the impact of $\alpha$ on temperature of the fluid. This variation $\alpha$ highlights the hidden properties of the fluid temperature in channel and explained the sensitive memory effect of the temperature during the dynamics of the fluid. This variation of $\alpha$ on temperature have been noted and can be applied many physical situations. Furthermore, the results obtained can be easily compare with experimental results by varying the values of $\alpha$ in the specified range. The comparison of the blood-based tri-hybrid nano-liquid ($\text{Fe}_{3} \text{O}_{4} + \text{Zn} + \text{Au}$\blood) with unitary nanofluid ($\text{Fe}_{3} \text{O}_{4}$\blood), (Zn\blood) and (Au\blood) is presented in Fig. 4. This comparative analysis help us to identify the thermal impact of each nanoparticles in the blood separately along with the tri-hybrid nano-liquids. From this analysis it is found that the blood-based tri-hybrid nano-liquids ($\text{Fe}_{3} \text{O}_{4} + \text{Zn} + \text{Au}$\blood) shows the effective thermal performance in the blood. Furthermore, the considered nanoparticles have its unique biomedical applications in blood. In the given study Zn is considered for decreasing the viscosity of the blood which is useful in COVID-19 disease, and gold Au is used to target the cancer cell in the human body. Figure 5 elucidates the impact of volume fraction of tri-hybrid nano-liquid $\phi_{hnf}$ on the fluid flow in channel. This figure capture the characteristic and behavior of volume fraction of tri-hybrid nano-liquid $\phi_{hnf}$ on the temperature profile. This shows that higher the concentration of $\phi_{hnf}$ from 0.01 to 0.04 the temeprature. From this it is worth noting that increasing the concentration within the fluid the resistive forces developed within the fluid which causes the increase in the kinetic energy as a result fluid temeprature rises in the channel. Moreover, by considering the concentration $\phi_{hnf} = 0$ implies that the blood have no nanoparticles and become conventional fluid. Figure 6 examines the involvement of $Re$ on the temperature of the fluid in channel. This figure clearly depict that the increasing values of $Re$ the temperature of the fluid can be control. Figure 7 depicts the impact of $Pr$ on the temeprature of the fluid. This variation in temeprature is due to the fact that increasing the values of $Pr$ results a decrease in the temeprature it is because of the increase in the thermal conductivity of the fluid due which the temperature of the couple stress Casson tri-hybrid nanofluid reduces.

**Figure 8:**
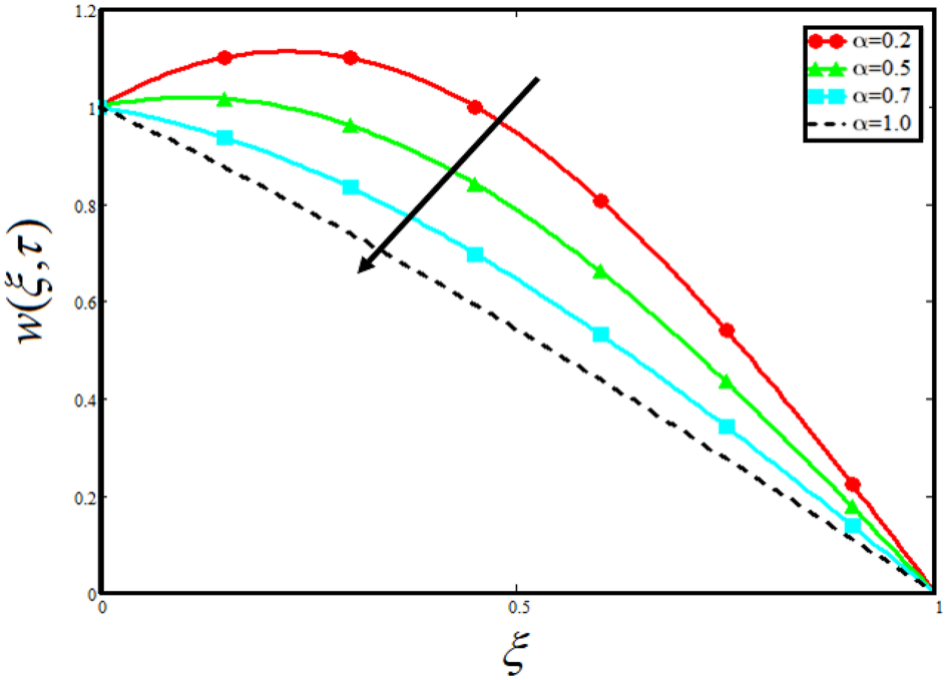
Variation in velocity of nanofluid against different values of $\alpha$ when $\tau = 1$, $\phi_{hnf} = 0.02$, $\Pr = 21$, $Gr = 4$, $G = 2$, $\lambda = 3$, $\beta = 1.2$ and ${\text{Re}} = 1.2$.

**Figure 9:**
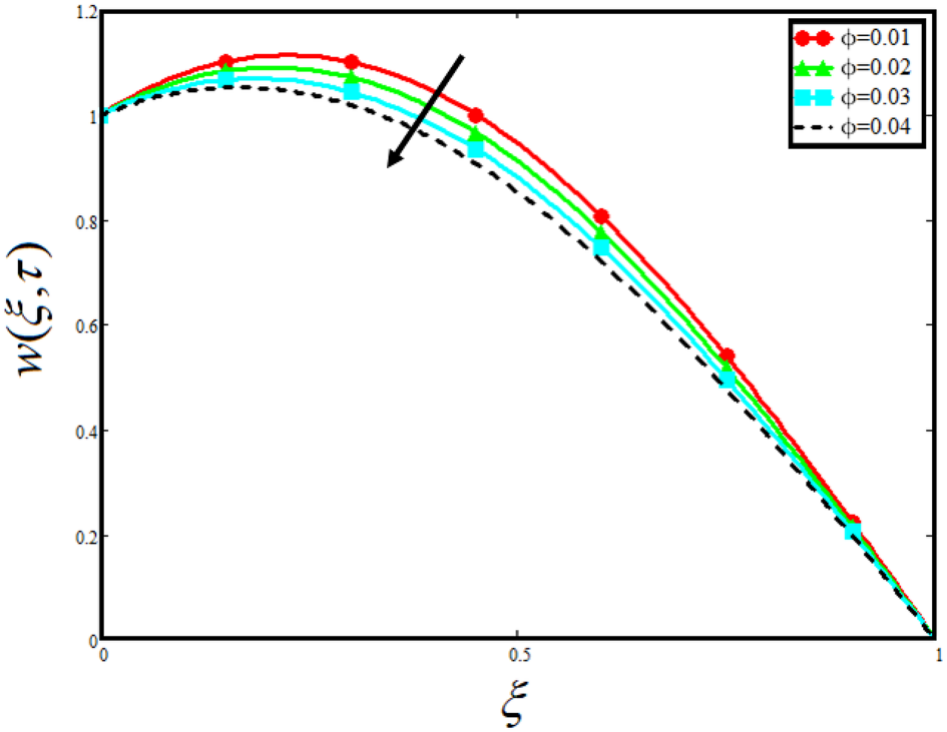
Variation in velocity of nanofluid against different values of $\phi_{hnf}$ when $\tau = 1$, $\alpha = 0.5$, $\Pr = 21$, $Gr = 4$, $G = 2$, $\lambda = 3$, $\beta = 1.2$ and ${\text{Re}} = 1.2$.

**Figure 10:**
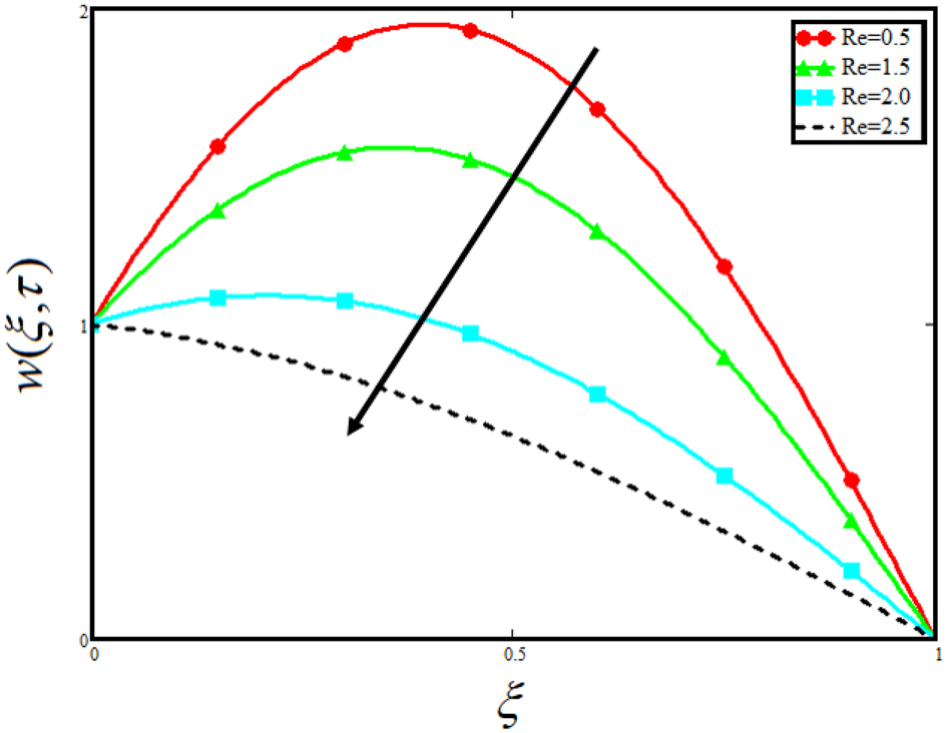
Variation in velocity of nanofluid against different values of ${\text{Re}}$ when $\tau = 1$, $\phi_{hnf} = 0.02$, $\Pr = 21$, $Gr = 4$, $G = 2$, $\lambda = 3$, $\beta = 1.2$ and $\alpha = 0.5$.

**Figure 11:**
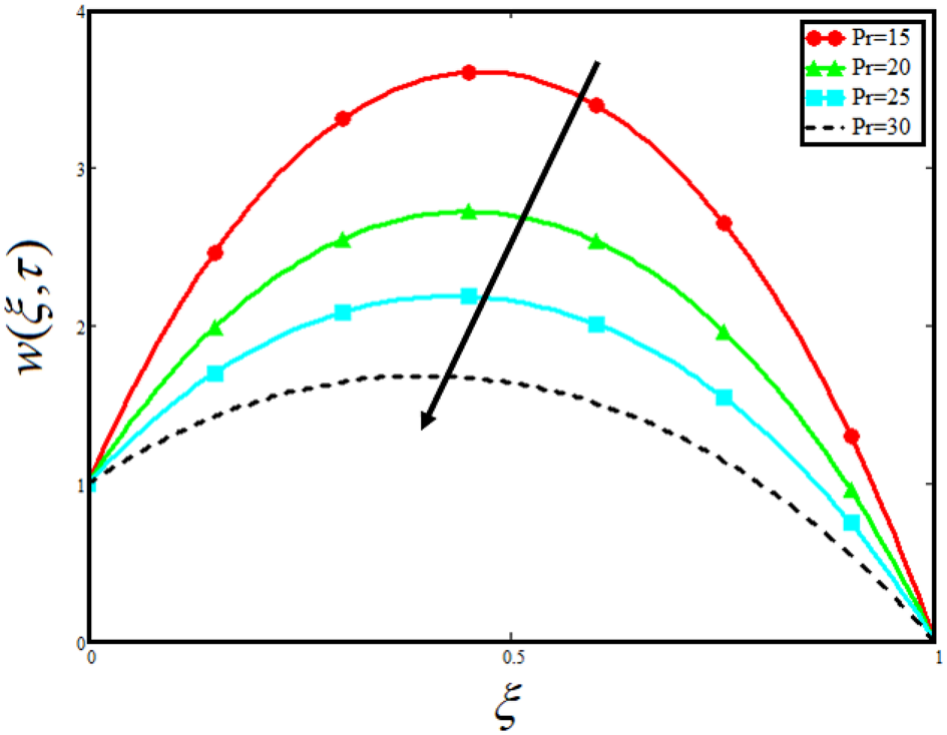
Variation in velocity of nanofluid against different values of $\Pr$ when $\tau = 1$, $\phi_{hnf} = 0.02$, $\alpha = 0.5$, $Gr = 4$ , $G = 2$, $\lambda = 3$, $\beta = 1.2$ and ${\text{Re}} = 1.2$.

**Figure 12:**
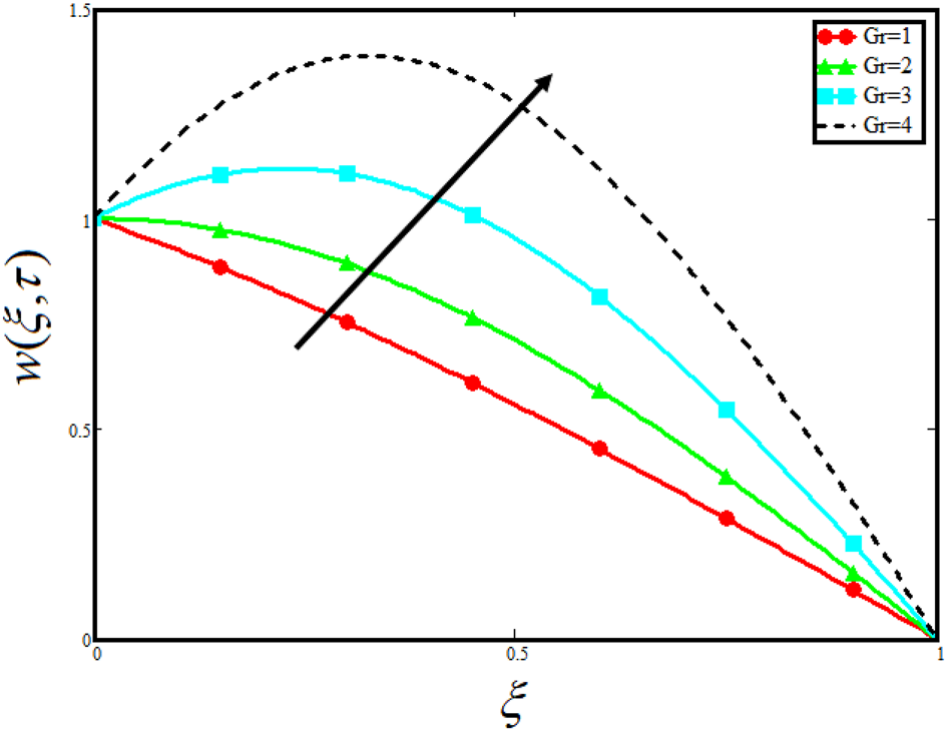
Variation in velocity of nanofluid against different values of $Gr$ when $\tau = 1$, $\phi_{hnf} = 0.02$, $\Pr = 21$, $\alpha = 0.5$, $G = 2$, $\lambda = 3$, $\beta = 1.2$ and ${\text{Re}} = 1.2$.

**Figure 13:**
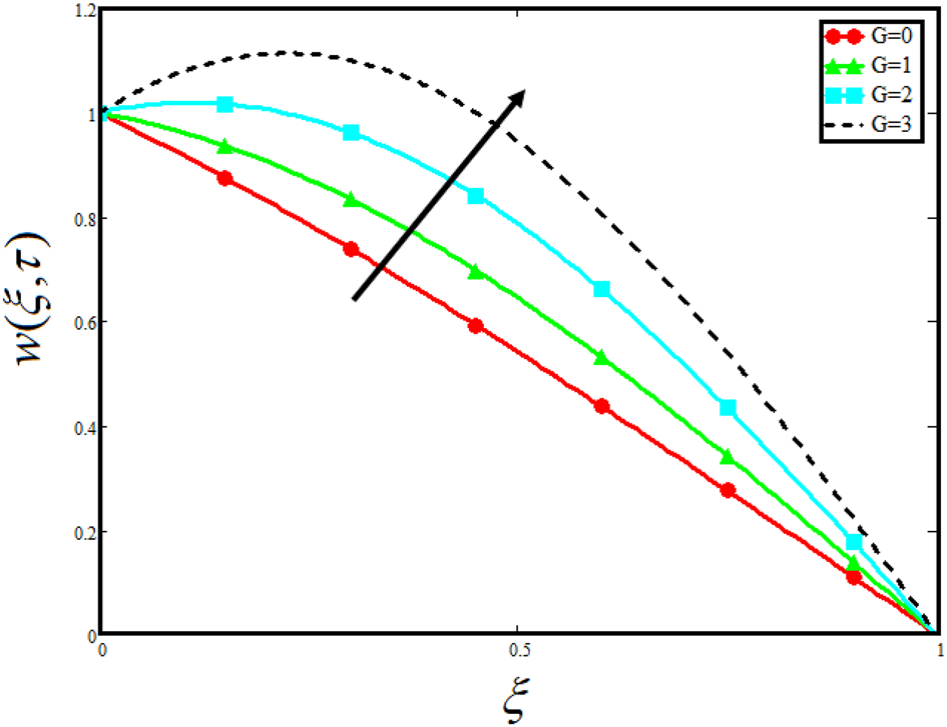
Variation in velocity of nanofluid against different values of $G$ when $\tau = 1$, $\phi_{hnf} = 0.02$, $\Pr = 21$, $Gr = 4$, $\alpha = 0.5$, $\lambda = 3$, $\beta = 1.2$ and ${\text{Re}} = 1.2$.

**Figure 14:**
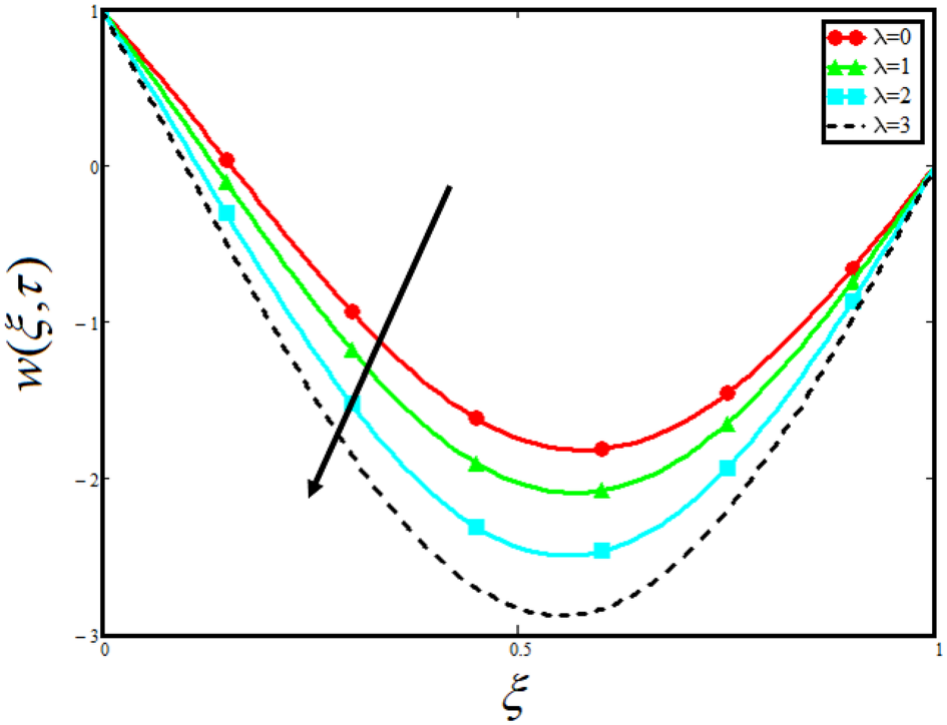
Variation in velocity of nanofluid against different values of $\lambda$ when $\tau = 1$, $\phi_{hnf} = 0.02$, $\Pr = 21$, $Gr = 4$, $G = 2$, $\alpha = 0.5$, $\beta = 1.2$ and ${\text{Re}} = 1.2$.

**Figure 15:**
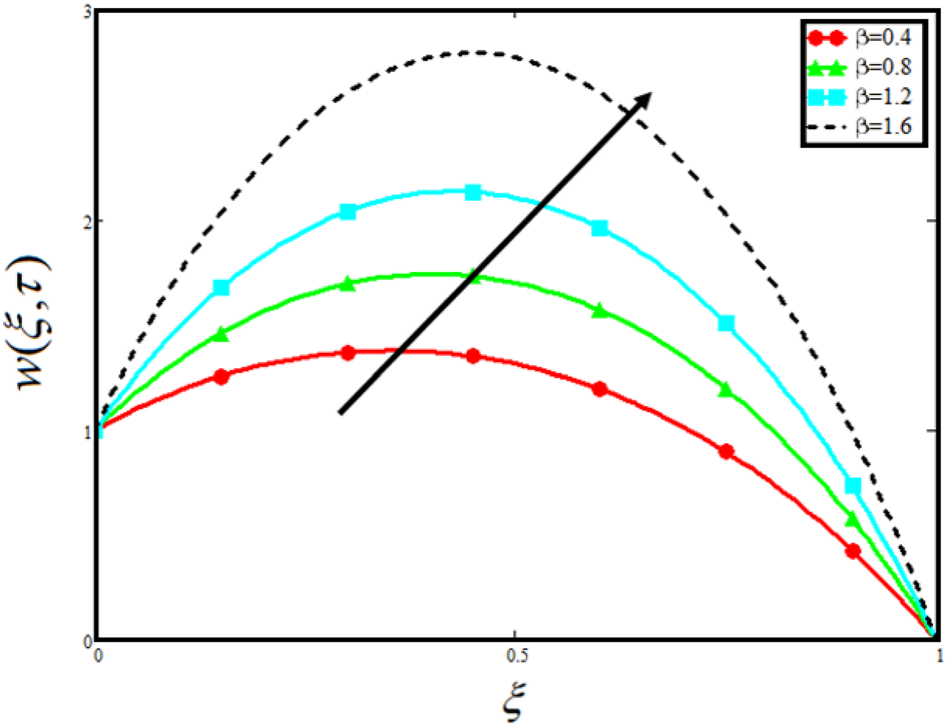
Variation in velocity of nanofluid against different values of $\beta$ when $\tau = 1$, $\phi_{hnf} = 0.02$, $\Pr = 21$, $Gr = 4$, $G = 2$, $\lambda = 3$, $\alpha = 0.5$ and ${\text{Re}} = 1.2$.

Figure 8 visualizes the impact of $\alpha$ on the fluid velocity and variation is noted. The reported profile for the fluid velocity predicts that velocity get lowered for higher values of $\alpha$. This physical exploration of the fluid velocity claimed that the higher values of $\alpha$ shows decline in the fluid velocity it is because $\alpha$ can explain the hidden sensitive memory of the system in the blood flow. Moreover, $\alpha$ predicts the best results for fluid velocity and maybe used in many experimental analysis for accuracy. The experimentalists can get the required results by changing the fractional constant between zero and one. Figure 9 identified the presence of volume friction of tri-hybrid nano-liquids $\phi_{hnf}$ for the physical applications in the blood. The reported profile of velocity in the present analysis claimed that slowdown in the motion of tri-hybrid nano-liquid flow occurs it is due to the fact that increasing the concentration of tri-nanoparticles in the blood developed the resistive forces which make them slowdown in the channel. The higher the concentration in the fluid increase the viscosity of the blood as a results the fluid velocity declines. For the purpose of comparison of the regular fluid with tri-hybrid nanofluid considering $\phi_{hnf} = 0$ which make them regular fluid and can be observed that heat transfer is higher in tri-hybrid nano-liquids as compare to unitary nano-liquids. Figure 10 indented the physical perspective of $Re$ on the velocity of tri-hybrid nano-liquid in channel. The physical exploration of $Re$ on velocity profile shows decline in the fluid velocity. This decline in the fluid motion in channel occurs due to the fact that an increment in the $Re$ cause higher the density of the fluid and hence fluid velocity declines. The physical perspective of the $Pr$ on the flow is highlighted in Fig. 11. The graphical sketch for the parameter $Pr$ on velocity of tri-hybrid nano-liquid claimed that larger values of $Pr$ decreases the fluid velocity. This decline in the fluid flow is due to the fact that for increment in $Pr$ results a decrease in the thermal conductivity of the working fluid which make the inter molecular kinetic energy reduces due to which the fluid flow retards in the boundary layer region and hence the fluid velocity slow down. Figure 12 claimed that higher values of $Gr$ make the fluid move faster in channel it is because increasing $Gr$ developed the boyancy forces within the fluid as a result the velocity increase as shown in figure. Figure 13 identified that velocity of the fluid get higher when increase in the magnitude of external pressure. This increment occur in the fluid velocity because the external pressure acting on the fluid in channel make them accelerated as a results velocity increases. Figure 14 claim that the velocity get lowered for higher values of $\lambda$ as shown. The velocity of the fluid declines with increment in $\lambda$ it is because the viscous forces become dominant as result the resistive forces get higher and hence the velocity of the fluid reduces in channel. Figure 15 presents the impact of Casson parameter $\beta$ on the fluid velocity. From the physical sketch it is illustrated that higher the $\beta$ causes an increase in the fluid motion in channel. This behavior of $\beta$ can highlight the dynamics of fluid flow in many physical applications and can be used in different physical situation which enable to describe the impact of $\beta$ on the fluid flow.

**Figure 16:**
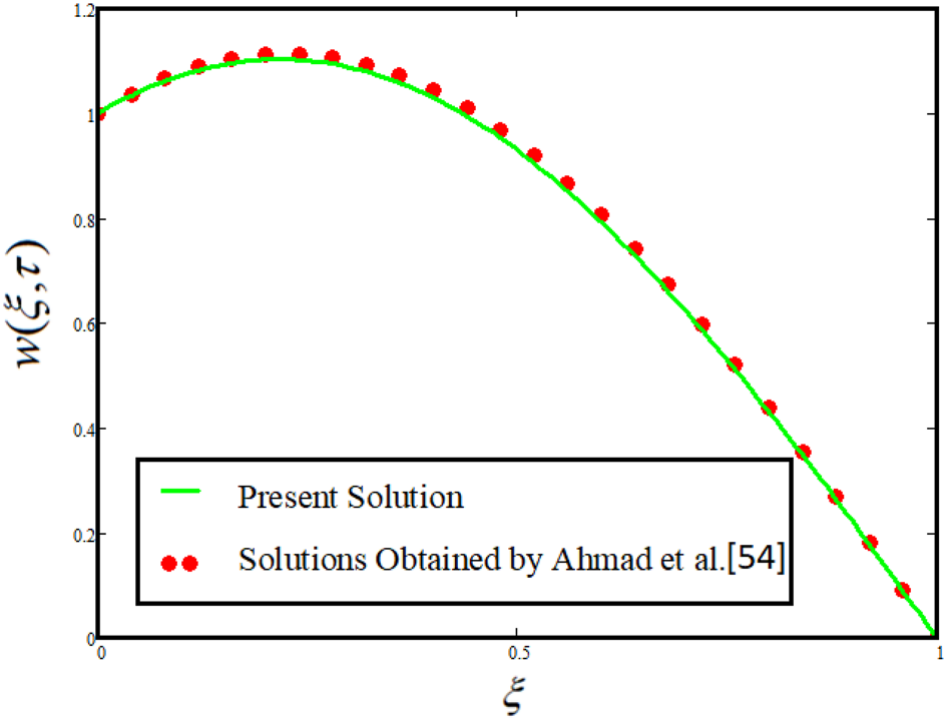
Comparison between the present solutions and Ahmad et al.^54^ when $\tau = 1$, $\phi_{hnf} = 0$, $\Pr = 21$, $Gr = 4$, $G = 0$, $\lambda = 3$, $\alpha = 0.5$ and ${\text{Re}} = 1.2$.

**Figure 17:**
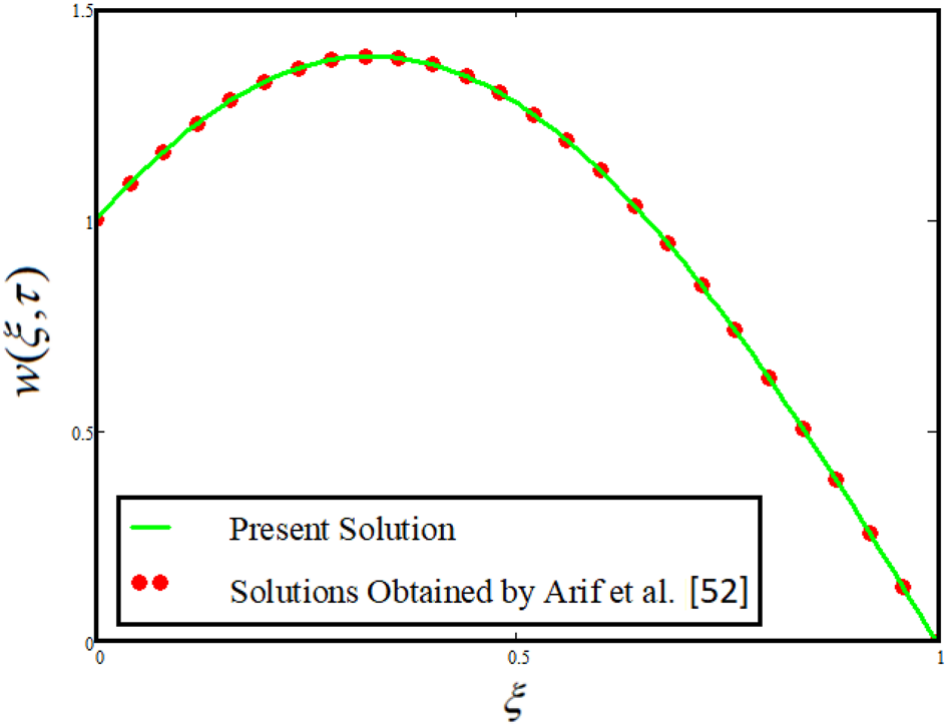
Comparison between the present solutions and Arif et al. [A] when $\tau = 1$, $\phi_{hnf} = 0$, $G = 1.2$, $\lambda = 3$, $\alpha = 0.5$ and ${\text{Re}} = 1.2$.

The comparative analysis between the present obtained solutions and solutions obtained by Ahmad et al.^54^ is highlighted in Fig. 16. From the figure it is worth noting that our solution shows a strong agreement with the published work which validate our results by neglecting the effect of external pressure and concentration of nanoparticles volume fraction. Similarly, Fig. 17 depicted that by incorportating Casson fluid parameter $\frac{1}{\beta } \to 0$, $\left( {Gr = 0} \right)$ and $\phi_{1} = \phi_{2} = \phi_{3} = 0$ then our obtained solutions reduced to the solution obtained by Arif et al.^52^ which verify our obtained results. The comparison is plotted in Fig. 17.

Table 1 provide the thermo-physical properties of the base fluid blood and different nanoparticles which are considered in the present analysis. The properties of blood, $\text{Fe}_{3} \text{O}_{4}$, $\text{Zn}$ and $\text{Au}$ are mentioned in the table. Table 2 shows the numerical values of the obtained solutions and the impact of various parameters are calculated in Nusselt number. The changes occur in the numerical values of Nusselt number can be seen from the table. From this tablet he numerical values have been calculated and noticed that higher $\alpha$ from 0.5 to 0.7 a decrease is noted. Following the same way for the higher values of time $\tau$ decline is noted, increasing $\phi_{hnf}$ the increment is occur in the Nusselt number, $Pr$ have shown increment while $Re$ shows the decline in the numerical values of Nusselt number. To calculate the percent enhancement in the Nusselt number may help in many engineering problems and they can get notice the efficiency of the present considered nanoparticles in the blood. For that reason the percentage enhancement is highlighted in Table 3 for varying the concentration of the tri-hybrid nanoparticles. The variation for $\phi_{hnf}$ is tabulated and presented. The results calculated in Table 3 for blood-based tri-hybrid nano-liquids have many physical aspects. In the present analysis spherical shaped ferric oxide $\left( {\text{Fe}_{3} \text{O}_{4} } \right)$, platelets shaped Zinc $(\text{Zn})$ and cylindrical shaped gold $\left( \text{Au} \right)$ have taken in blood for biomedical applications. From the calculated numerical values for tri-hybrid nanoparticles the rate can be enhanced up-to 8.05%. Next we have calculated the variation in heat transfer rate for unitary nanofluid in blood. Table 4 describes the percentage enhancement in the heat which is shown for different volume fraction of spherical shaped $\left( {\text{Fe}_{3} \text{O}_{4} } \right)$ in the blood and found that it enhances the rate of heat transfer up-to 4.630%. Table 5 shows the percentage enhancement for different volume fraction of platelet shaped $\left( \text{Zn} \right)$ nanoparticles in the blood and found that it enhances the rate of heat transfer up-to 8.984%. Table 6 shows the percentage enhancement for different volume fraction of cylindrical shaped $\left( \text{Au} \right)$ nanoparticles in n the blood and found that it enhances the rate of heat transfer up-to 10.407%.

**Table 2:** Nusselt number variation against different parameters.

$\alpha$	$\tau$	$\phi_{hnf}$	$\Pr$	${\text{Re}}$	$Nu$
0.5	1	0.02	21	1.2	4.898
**0.7**	1	0.02	21	1.2	4.434
0.5	**1.5**	0.02	21	1.2	4.594
0.5	1	**0.03**	21	1.2	4.993
0.5	1	0.02	**22**	1.2	5.013
0.5	1	0.02	21	**1.5**	5.476

Significant values are in bold.

**Table 3:** Percentage enhancement in Nusselt number.

$\phi_{hnf}$	$\alpha$	$\tau$	$\Pr$	${\text{Re}}$	$Nu$	Percentage
0.00	0.5	1	21	1.2	4.708	–
0.01	0.7	1	21	1.2	4.804	2.039
0.02	0.5	1.5	21	1.2	4.898	4.035
0.03	0.5	1	21	1.2	4.993	6.053
0.04	0.5	1	25	1.2	5.087	8.05

**Table 4:** Percentage enhancement for different volume fraction of Fe_3_O_4_.

$\phi (\text{Fe}_{3} \text{O}_{4} )$	$\alpha$	$\tau$	$\Pr$	${\text{Re}}$	$Nu$	Percentage
0.00	0.5	1	21	1.2	4.708	–
0.01	0.7	1	21	1.2	4.762	1.147
0.02	0.5	1.5	21	1.2	4.817	2.315
0.03	0.5	1	21	1.2	4.871	3.462
0.04	0.5	1	25	1.2	4.926	4.630

**Table 5:** Percentage enhancement for different volume fraction of Zn.

$\phi (\text{Zn})$	$\alpha$	$\tau$	$\Pr$	${\text{Re}}$	$Nu$	Percentage
0.00	0.5	1	21	1.2	4.708	–
0.01	0.7	1	21	1.2	4.815	2.272
0.02	0.5	1.5	21	1.2	4.921	4.524
0.03	0.5	1	21	1.2	5.026	6.754
0.04	0.5	1	25	1.2	5.131	8.984

**Table 6:** Percentage enhancement for different volume fraction of Au.

$\phi (\text{Au})$	$\alpha$	$\tau$	$\Pr$	${\text{Re}}$	$Nu$	Percentage
0.00	0.5	1	21	1.2	4.708	–
0.01	0.7	1	21	1.2	4.833	2.655
0.02	0.5	1.5	21	1.2	4.956	5.267
0.03	0.5	1	21	1.2	5.078	7.858
0.04	0.5	1	25	1.2	5.198	10.407

## Conclusion

In this study, the significance of tri-hybrid nano-liquids are highlighted and discussed its uses in many physical situations. In the present work, the mixture of nanoparticles formed in a single base fluid blood for the applications in biological sciences. It is found experimentally, that the working capacity of tri-hybrid nano-liquids is higher compared to unitary nanofluids. Moreover, the shape factor is also play a key role in the thermal transport phenomena. Although nanofluid have good working capability in many applied sciences but they need to be further improved due to that reason the mixture of different nanoparticles in single base fluid is considered in the present analysis. It is worth noting that the various shaped of three different kinds of nanoparticles are considered in the blood and name as “tri-hybrid nanofluid”. In this study the blood-based tri-hybrid nanofluids with various shaped spherical, platelet and cylindrical shape nanoparticles for the purpose of biomedical applications have been considered. The combination of spherical shaped ferric oxide $\left( {\text{Fe}_{3} \text{O}_{4} } \right)$, platelets shaped Zinc $(\text{Zn})$ and cylindrical shaped gold $(\text{Au})$ is taken in the blood for biomedical applications. The suspension of platelet shaped $\left( \text{Zn} \right)$ is used in the blood for decreasing viscosity of the blood which has useful applications in COVID-19 infectious disease. If a $(\text{Zn})$ nanoparticle is injected in the blood of COVID-19 infected patient it will decrease the viscosity of the blood and prevent the patient from the blood clotting. In addition zinc nanoparticles use as preventive therapeutic agent and increase the immune response against COVID-19 infectious disease. Similarly, cylindrical shaped gold $\left( \text{Au} \right)$ nanoparticles have useful applications in the cancer therapy which is used in the blood to hit the cancer cells in the body.

The ferric oxide $\text{Fe}_{3} \text{O}_{4}$ nanoparticles is used to target the anticancer drug delivery systems. Keeping the above motivation in mind the present study considered blood flow in channel with the dispersion of three different kinds of nanoparticles. The model is formulated and presented as momentum and energy equations in terms of PDE’s along with IC’s and BC’s. The present model is generalized by developing AB-fractional derivatives. The closed form results are evaluated by considering tha application of integral transforms (Laplace and Fourier. For the physical exploration of the obtained results MATHCAD software is used. All the parameters are physically explored and highlight through graphs for clear understanding in the fluid dynamics. Moreover, the Nusselt number and percentage enhancement of tri-hybrid nanofluids, spherical shaped $\left( {\text{Fe}_{3} \text{O}_{4} } \right)$, platelets shaped $(\text{Zn})$ and cylindrical shaped $(\text{Au})$ is calculated and presented in tables.

Let us underling the main results provided within the present analysis:

- we discovered that using blood-based tri-hybrid nano-liquid enhances heat transfer up-to 8.05% of the blood.
- we have shown that spherical shaped $\left( {\text{Fe}_{3} \text{O}_{4} } \right)$ enhances the thermal transport up-to 4.63%.
- we demonstrated that by suspending the platelets shaped $(\text{Zn})$ in the blood enhances the thermal transport up-to 8.984%.
- we have shown that the uniform dispersion of cylindrical shaped $(\text{Au})$ enhances the thermal transport up-to 10.407% in the blood.
- we provided evidences showing that the temperature of the fluid get higher with the increase in $\alpha$ and volume friction $\phi_{hnf}$.

Moreover, we have also shown that

- the temperature distribution get lower for higher values of $Re$ and $Pr$.
- the velocity get higher for increasing values of $Gr$, $G$ and $\beta$ .
- the fluid velocity get slower for greater values of $\alpha$, $\phi_{hnf}$, $Re$, $Pr$ and $\lambda$.

## Future suggestions

The idea in the present analysis can be extended in future as:

- One can extend this work by changing the boundary conditions like, ramped wall temperature, slip boundary conditions.
- One can choose different base fluid like, engine oil, honey, kerosene oil, transformer oil etc.,
- In future one can choose different nanoparticles for different scientific purposes.
- This study can be further extend by changing the geometries like one can investigate the idea of ternary hybrid nanofluid by using stretching sheet, rotating disc, inclined plat and channel etc.
- in different boundary conditions
- This idea can be used in engineering applications.
- This classical model can be transformed by applying Caputo, Caputo-Fabrizio and fractal-fractional derivatives.

## Data Availability

All data used in this manuscript have been presented within the manuscript. No data is hidden or restricted.

## List of symbols

$$U_{0}$$: Constant velocity $$\left[ {\text{m/s}} \right]$$
$$t$$: Time $$\left[ {\text{s}} \right]$$
$$u$$: Velocity $$\left[ {\text{m/s}} \right]$$
$$T$$: Temperature $$\left[ {\text{K}} \right]$$
$$g$$: Gravitational acceleration $$\left[ {{\text{m/s}}^{2} } \right]$$
$h$: Channel height between two parallel plates $\left[ {\text{m}} \right]$
$\alpha$: Atangana–Baleanu fractional operator
$\beta$: Casson fluid parameter
$\text{Fe}_{3} \text{O}_{4}$: Ferric oxide nanoparticles
$$Zn$$: Zinc nanoparticles
$$Au$$: Gold nanoparticles
$$\Pr$$: Prandtl number
$Gr$: Dimensionless parameter Grashof number
$$\theta$$: Temperature $$\left[ {\text{K}} \right]$$
$^{AB} {\mathbf{D}}_{\tau }^{\alpha } (.)$: Atangana–Baleanu fractional derivatives
$F_{\alpha } (.,.)$: Robotnov and Hartleys' function
$$N(\alpha )$$: Normalization function
$$Nu$$: Nusselt number
$\rho_{hnf}$: Tri-hybrid nanofluid density $\left[ {{\text{kg/m}}^{3} } \right]$
$\mu_{hnf}$: Tri-hybrid nanofluid viscosity $\left[ {{\text{N}}\;{\text{s}}/{\text{m}}^{2} } \right]$
$k_{hnf}$: Tri-hybrid nanofluid thermal conductivity $\left[ {{\text{W}}/{\text{m}}\;{\text{K}}} \right]$
$\phi_{hnf}$: Volume friction of the tri-hybrid nanoparticles
$$C_{p}$$: Heat capacity $$\left[ {{\text{J}}/{\text{kg}}\;{\text{K}}} \right]$$
$\beta_{T}$: Coefficient of thermal expansion $\left[ {1/{\text{K}}} \right]$
$$H(t)$$: Heaviside function
$T_{w}$: Temperature of the wall $\left[ {\text{K}} \right]$
$$T_{h}$$: Ambient temperature $$\left[ {\text{K}} \right]$$
$\phi_{1}$: Volume fraction of $\text{Fe}_{3} \text{O}_{4}$ nanoparticles
$\phi_{2}$: Volume fraction of Zn nanoparticles
$\phi_{3}$: Volume fraction of Au nanoparticles
$$\Psi$$: Sphericity
$$w$$: Dimensionless velocity $$\left[ {\text{m/s}} \right]$$
$$\tau$$: Dimensionless time $$\left[ {\text{s}} \right]$$
$E_{\alpha }$: Mittag–Leffler function
$\lambda$: Couple stress fluid parameter
