# Author Correction: Heat transfer analysis of fractional model of couple stress Casson tri-hybrid nanofluid using dissimilar shape nanoparticles in blood with biomedical applications

**DOI:** 10.1038/s41598-023-35419-7

**Published:** 2023-05-30

**Authors:** Muhammad Arif, Luca Di Persio, Poom Kumam, Wiboonsak Watthayu, Ali Akgül

**Affiliations:** 1 Fixed Point Research Laboratory, Fixed Point Theory and Applications Research Group, Center of Excellence in Theoretical and Computational Science (TaCS-CoE), Faculty of Science, King Mongkut’s University of Technology Thonburi (KMUTT), 126 Pracha Uthit Rd., Bang Mod, Thung Khru, Bangkok, 10140 Thailand; 2 Center of Excellence in Theoretical and Computational Science (TaCS-CoE), Faculty of Science, King Mongkut’s University of Technology Thonburi (KMUTT), 126 Pracha Uthit Rd., Bang Mod, Thung Khru, Bangkok, 10140 Thailand; 3 Department of Computer Science, College of Mathematics, University of Verona, Verona, Italy; 4 Department of Medical Research, China Medical University Hospital, China Medical University, Taichung, 40402 Taiwan; 5 Department of Mathematics, Faculty of Arts and Sciences, Siirt University, 56100 Siirt, Turkey; 6 Department of Computer Science and Mathematics, Lebanese American University, Beirut, Lebanon; 7 Mathematics Research Center, Department of Mathematics, Near East University, Near East Boulevard, PC: 99138, Nicosia /Mersin 10, Nicosia /Mersin, Turkey

Correction to: *Scientific Reports* 10.1038/s41598-022-25127-z, published online 21 March 2023

The original version of this Article contained an error in the Acknowledgments section.

“The authors acknowledge the financial support provided by the Center of Excellence in Theoretical and Computational Science (TaCS-CoE), KMUTT. Moreover, this research project is supported by Thailand Science Research and Innovation (TSRI) Basic Research Fund: Fiscal year 2022 (FF65). The first author Muhammad Arif appreciate the support provided by Petchra Pra Jom Klao Ph.D. Research Scholarship (Grant No. 14/2562 and Grant No. 25/2563).”

now reads:

“The authors acknowledge the financial support provided by the Center of Excellence in Theoretical and Computational Science (TaCS-CoE), KMUTT. Moreover, this research project is supported by Thailand Science Research and Innovation (TSRI) Basic Research Fund: Fiscal year 2023 under project number FRB660073/0164. The first author Muhammad Arif appreciates the support provided by Petchra Pra Jom Klao Ph.D. Research Scholarship (Grant No. 14/2562 and Grant No. 25/2563).”

The original Article has been corrected.

